# Risk of bias and reporting completeness of randomised controlled trials in burn care: protocol for a systematic review

**DOI:** 10.1136/bmjopen-2019-033472

**Published:** 2019-12-18

**Authors:** Amber Young, Barnaby C Reeves, Hung-Yuan Cheng, Jason Wasiak, Duncan Muir, Anna Davies, Jane Blazeby

**Affiliations:** 1 Bristol Centre for Surgical Research, Population Health Sciences, Bristol Medical School, University of Bristol, Bristol, UK; 2 Paediatric Anaesthesia, University Hospitals Bristol NHS Foundation Trust, Bristol, UK; 3 Bristol Trials Centre (BRI-Hub), Bristol Medical School, University of Bristol, Bristol, UK; 4 Population Health Sciences, Bristol Medical School, University of Bristol, Bristol, UK; 5 Olivia Newton John Cancer Wellness & Research Centre, Department of Radiation Oncology, Austin Health, Heidelberg, Victoria, Australia; 6 Austin Health Clinical School of Nursing, Latrobe University, Heidelberg, Victoria, Australia; 7 Centre for Academic Child Health, University of Bristol, Bristol, UK; 8 NIHR Biomedical Research Centre, University of Bristol and University hospitals Bristol NHS Foundation Trust, Bristol, UK

**Keywords:** bias, burns, cochrane, consort, quality, randomised controlled trials as topic

## Abstract

**Introduction:**

Burn care represents a healthcare and economic burden to patients internationally. Choice of the most clinically effective treatment strategies requires evidence which is best obtained through high-quality randomised controlled trials (RCT). The number of published RCTs of burn care is increasing. However, trial quality and reporting standards are unclear. This study will assess the risk of bias and adequacy of reporting in recent burn care RCTs using tools endorsed by the Cochrane Collaboration.

**Methods and analysis:**

A systematic literature review will be undertaken, assessing parallel group RCTs evaluating therapeutic interventions for patients with cutaneous burns. Literature searches will use Ovid Medline, Ovid Embase, Web of Science and the Cochrane Library. Separate searches for each database will include medical subject heading and free text terms including ‘burn’, ‘scald’, ‘thermal injury’ and ‘RCT’. Two reviewers will independently assess each study for inclusion. Risk of bias (RoB) will be assessed with the revised tool (RoB 2) and reporting completeness with the CONsolidated Standards of Reporting Trials (CONSORT) 2010 guidelines. We will report a narrative synthesis of all studies, including domain specific, and overall risk of bias for the primary outcome of each trial. Inter-rater agreement for RoB 2 will be reported using Fleiss’s Kappa. For adherence to the CONSORT guidelines, we will generate a completeness of reporting index for the ﬁve domains.

**Ethics and dissemination:**

No ethics approval is required because published documents will be used. Findings of the study will be disseminated in a peer-reviewed journal and presented at conferences.

**PROSPERO registration number:**

CRD42018111020.

**Strengths and limitations of this study**

- This systematic review will be the first to assess both the internal validity of burn care randomised controlled trials using the Risk of Bias 2 (RoB 2) tool and adherence to CONsolidated Standards of Reporting Trials 2010 reporting guidelines.
- The inter-rater reliability of RoB 2 will be assessed using six reviewers from different research backgrounds to simulate real-world conditions.
- The systematic review will be limited to 5 years and to articles published in English.
- Assessments of external validity will not be performed.

## Introduction

In 2009, Chalmers and colleagues estimated clinical research waste to be 85% of global investment.^1^ Wastage was attributed to methodological flaws in randomised controlled trials (RCTs). Poor quality RCTs may result in misleading conclusions increasing research waste.^2–4^ Numbers of RCTs in burn care research are increasing, as new technologies are regularly introduced.^5–9^ However, trial quality and adherence to reporting standards are uncertain.^10–13^ Previous studies are limited by their lack of comprehensiveness and methods of quality assessment.^14–16^ Quality assessment of RCTs involves evaluating internal and external validity. External validity assesses whether study results can be generalised to other populations.^17^ Internal validity, or risk of bias (RoB), is the extent to which the study design is free from bias; a systematic error that leads to a deviation of results from the truth.^18^ Transparent reporting is necessary to allow a clear assessment of the trial design and conduct as recommended by the Cochrane Collaboration.^19 20^

Using an objective method to assess RoB and reporting completeness has become increasingly common and considered good practice.^18 21^ For assessment of RoB, many tools are available, including overall scores and checklists.^22 23^ The Cochrane collaboration endorse a different type of tool based on individual domains.^18^ RoB domains are assessed separately, with the overall RoB formed relating to the highest domain score. Accumulating evidence shows that this is the best tool for assessing the RoB of RCTs.^24–26^ A revised version of the tool (RoB 2) has been developed which now examines single trial outcomes, includes ‘signalling questions’ to aid decision-making and a method to allow an overall RoB judgement.^27^ As yet, this tool has not been tested for inter-rater reliability. The Cochrane collaboration recommend ‘The CONsolidated Standards of Reporting Trials (CONSORT) statement’ to assess adherence to reporting standards.^28^

The aim of this review is to assess the risk of bias and adherence to reporting standards using Cochrane-approved tools. It will also assess inter-rater reliability of the new RoB 2 tool.

## Methods

This review will meet its aim with these objectives. It will:

- Determine the number of parallel-group, individually randomised trials assessing burn care interventions published over the last 5 years and retrieve included full-text articles.
- Assess the internal validity of the included RCTs using the revised Cochrane-endorsed RoB 2 tool.
- Assess the inter-rater reliability for the new RoB 2 tool.
- Assess the adherence of the included RCTs to CONSORT 2010 reporting guidelines.

### Literature review search strategy

The systematic review will adhere to this prespecified protocol and the Preferred Reporting Items for Systematic Reviews and Meta-Analyses (PRISMA) statement. This protocol will be aligned to the PRISMA-P statement.^29–31^ It has been registered with the PROSPERO international prospective register of systematic reviews. We will report any amendments to the protocol that occur while undertaking the study, within the final manuscript.

#### Study eligibility

##### Types of studies

Included studies must be full-text individually randomised parallel RCTs published in peer-reviewed journals limited to those allocating human subjects to an intervention or control group. We have planned to limit the search to the last 5-year period. We will not attempt to include all burn care RCTs, as would be undertaken in assessment of the effect of an intervention. Instead, we will investigate whether recent publications of burn care RCTs adhere to the CONSORT statement when reporting their findings and whether the trials we include are at low risk of bias according to the revised Cochrane RoB 2. We will exclude RCT protocols, conference proceedings, abstracts, non-English language publications, interim analysis reports and studies not involving human subjects. Health economic evaluation reports of clinical trials will be considered if they contain enough information for assessing risk of bias (eg, clearly described methods for the trial conduct). We will also exclude trials that compare treatments within subjects as there is not yet a RoB 2 tool designed for assessing such trials. Also, in burn care research, these are typically not cross-over trials as generally defined.^32 33^ Instead, they commonly use two wounds or two parts of the same wound. It is uncertain what the dependence between these might be or how treatments might ‘contaminate’ one another.

##### Types of participants

We will include studies evaluating two or more interventions in patients of any age with cutaneous burns. Studies where the population consists of patients with combined burn and mechanical injuries will be excluded, as the data relating to burn patients alone are likely to be difficult to disaggregate.

##### Type of interventions

Interventions to treat cutaneous burns of any aetiology.

##### Types of outcome

Clinical or patient-reported outcomes. Laboratory studies will be excluded.

### Identification of studies

A predefined search strategy previously designed by the authors in conjunction with experienced systematic reviewers to identify RCTs in the field of burn care will be used. Electronic searches of Ovid Medline, Ovid Embase, Web of Science and The Cochrane Library will be searched using medical subject heading and free-text terms including ‘burn’, ‘scald’ ‘thermal injury’ and ‘RCT’. To limit the search to RCTs, we will use terms derived from published RCT search strategies on Medline and the BMJ best practice guideline.^34 35^ The thesaurus vocabulary of each database will be used to adapt the search terms. The search strategy for Ovid Medline is included in a previous publication and in Table 1.^36^

Box 1 **Ovid Medline search strategy**

Ovid Medline search strategy

- Burns/(MESH) exp
- Burn*.tw
- Scald*.tw
- Thermal* adj injur*.mp
- or/1–4
- Heartburn.mp
- Burnout.mp
- (Burn* adj out).mp
- Burning.mp
- Burnetii.mp
- Burnish*.mp
- Burnet*.mp
- 6 OR 7 OR 8 OR 9 OR 10 OR 11 OR 12
- 5 NOT 13
- Limit to RCT, clinical trial, English language, human, last five years

### Study selection process

Before screening abstracts and full-text papers, authors undertaking study selection, RoB 2 assessment and data extraction, will undergo training to ensure a comparable understanding of the purpose of the review and the eligibility criteria. The reference management software EndNote (Endnote X8 Clarivate Analytics) will be used to compile all titles derived from the initial searches, with duplicates removed, for the review authors to screen titles and abstracts against the eligibility criteria. Screening of titles and abstracts will be completed by one reviewer (AY). Of these, 20% will be checked by another reviewer independently (DM). Any studies appearing to meet the inclusion criteria based on the abstract will be retrieved as full-text articles. Two reviewers will then read the full-text articles in their entirety to assess for eligibility, with decisions on inclusion and exclusion recorded (AY,DM). Reasons for exclusion will be ordered hierarchically from most to least important (box 2) and applied to each full-text paper. The most important reason for exclusion met by a paper will be recorded as the reason for exclusion. Any disagreements will be discussed with senior researchers (JB, BR).

Box 2 **Reasons for full-text exclusion**

- Duplicate.
- Not published within time period.
- Population not consisting of burn patients alone.
- Not a parallel group RCT.
- Not in English.
- Non-human/animal study.
- No full text available.
- Laboratory-based study.
- Volunteer study.
- Oesophageal burns only.
- Ocular burns only.
- Anaesthetic/sedation technique only (pain management included).
- Smoke inhalation injuries without an associated cutaneous burn.
- Diagnostic test trial.
- Protocol only.

### Data extraction and analysis

All studies will be assessed by two reviewers independently and then in duplicate (AY, DM), with disagreements resolved by discussion until consensus is reached or by a senior reviewer (BR). Data will be extracted into a standardised data extraction Microsoft Excel spreadsheet which will be specifically designed for this study. It will be adapted from the RoB 2 tool to include details for the CONSORT checklist by one of the coauthors (H-YC). Before starting the assessment, reviewers will undergo training with the spreadsheet. The reviewers will then independently pilot test each tool on five RCTs. If there are significant differences in the application of the tools in the pilot round, additional testing will be undertaken.^37^

Data extracted will include study details and research design. Study details will include author, publication year, number of sites and number of participants recruited per trial, design (full RCT, pilot study) and intervention tested. Risk of bias judgement will be reported for all trial primary outcomes at domain and overall level. Completeness of reporting will be reported for all RCTs.

### Assessment of risk of bias

Included studies will be objectively assessed for internal validity using RoB 2.^38^ The tool has five domains to assess bias arising from randomisation, deviations from intended interventions, missing outcome data, outcome measurement and in selection of the reported result. The assessment is specific to a single trial outcome. Categories of the overall risk of bias for the study outcome are low (risk of bias is low for all domains), some concerns (some concerns in at least one domain) and high (high risk of bias for at least one domain or some concerns for multiple domains).

#### Choice of outcome

Risk of bias will be assessed for the treatment effect for the primary outcome in each included trial. If the primary outcome is not reported explicitly, we will use the following decision rule to select the treatment effect to report: we will assess the treatment effect for the outcome used to calculate sample size and, if this too is not reported, assess the treatment effect for the outcome named in the title, then the first reported outcome in the results.^37^

#### Assessment of the inter-rater agreement of the RoB 2 tool

As RoB 2 is new, measuring agreement between reviewers will help assess whether the new guidance with signalling questions has improved the reliability of the tool compared with the previous version.^39–43^ To evaluate the inter-rater agreement, six independent reviewers will assess the same 30 studies allocated in a balanced incomplete blocks design (please see Appendix A), ensuring that each study is rated 3 times, each assessor rates 15 studies and all pairs of assessors rate 6 studies. Inter-rater agreement will be measured for each domain of bias and for the overall RoB judgement by calculating Fleiss’s Kappa scores.^44 45^ We will categorise agreement as poor (0.00), slight (0.01–0.20), fair (0.21–0.40), moderate (0.41–0.60), substantial (0.61–0.80) or almost perfect (0.81–1.00).^26^

## Assessment of reporting completeness

Completeness of reporting for each included RCT will be assessed using the latest revision of the CONSORT statement checklist.^46^ For assessing completeness of reporting, we will calculate a reporting index defined as the percentage of items reported in each of ﬁve domains (title/abstract, introduction, methods, results and discussion), as has been done previously.^47^

### Patient and public involvement

There was no patient or public involvement in designing the study or writing up the study protocol.

10.1136/bmjopen-2019-033472.supp1 Supplementary data

## Supplementary Material

Reviewer comments

Author's manuscript

## References

[1] Chalmers I, Glasziou P Avoidable waste in the production and reporting of research evidence. The Lancet 2009;374:86–9. 10.1016/S0140-6736(09)60329-9 19525005

[2] Yordanov Y, Dechartres A, Porcher R, et al Avoidable waste of research related to inadequate methods in clinical trials. BMJ 2015;350:h809 10.1136/bmj.h809 25804210 PMC4372296

[3] Ioannidis JPA, Greenland S, Hlatky MA, et al Increasing value and reducing waste in research design, conduct, and analysis. The Lancet 2014;383:166–75. 10.1016/S0140-6736(13)62227-8 PMC4697939 24411645

[4] Jüni P, Altman DG, Egger M Assessing the quality of controlled clinical trials. Bmj 2001;323:42–6. 11440947 10.1136/bmj.323.7303.42 PMC1120670

[5] Jeschke MG, Finnerty CC, Shahrokhi S, et al Wound coverage technologies in burn care: novel techniques. J Burn Care Res 2013;34:612–20. 10.1097/BCR.0b013e31829b0075 23877140 PMC3819403

[6] Selig HF, Lumenta DB, Giretzlehner M, et al The properties of an “ideal” burn wound dressing – What do we need in daily clinical practice? Results of a worldwide online survey among burn care specialists. Burns 2012;38:960–6. 10.1016/j.burns.2012.04.007 22571855

[7] Jeschke MG, Herndon DN Burns in children: standard and new treatments. The Lancet 2014;383:1168–78. 10.1016/S0140-6736(13)61093-4 PMC7859869 24034453

[8] Shahrokhi S, Arno A, Jeschke MG The use of dermal substitutes in burn surgery: acute phase. Wound Repair Regen 2014;22:14–22. 10.1111/wrr.12119 PMC3884830 24393152

[9] Young AE, Davies A, Bland S, et al Systematic review of clinical outcome reporting in randomised controlled trials of burn care. BMJ Open 2019;9:e025135 10.1136/bmjopen-2018-025135 PMC6398699 30772859

[10] Norman G, Christie J, Liu Z, et al Antiseptics for burns. Cochrane Database Syst Rev 2017;10 10.1002/14651858.CD011821.pub2 PMC6483239 28700086

[11] Breederveld RS, Tuinebreijer WE Recombinant human growth hormone for treating burns and donor sites. Cochrane Database Syst Rev 2012;12. 10.1002/14651858.CD008990.pub2 23235668

[12] Barajas-Nava LA, Lopez-Alcalde J Roqué I Figuls M, et al. antibiotic prophylaxis for preventing burn wound infection. Cochrane database of systematic reviews 2013;6. 10.1002/14651858.CD008738.pub2 PMC11303740 23740764

[13] Wasiak J, Cleland H, Campbell F, et al Dressings for superficial and partial thickness burns. 2013;18 10.1002/14651858.CD002106.pub4 PMC7065523 23543513

[14] Danilla S, Wasiak J, Searle S, et al Methodological quality of randomised controlled trials in burns care. A systematic review. Burns 2009;35:956–61. 10.1016/j.burns.2009.04.031 19545949

[15] Al-Benna S, Alzoubaidi D, Al-Ajam Y Evidence-Based burn care—An assessment of the methodological quality of research published in burn care journals from 1982 to 2008. Burns 2010;36:1190–5. 10.1016/j.burns.2010.03.011 20621705

[16] Sinha S, Yoon G, Shin W, et al Burn clinical trials: a systematic review of registration and publications. Burns 2018;44:263–71. 10.1016/j.burns.2017.11.001 29169699

[17] Rothwell PM External validity of randomised controlled trials: “To whom do the results of this trial apply?”. The Lancet 2005;365:82–93. 10.1016/S0140-6736(04)17670-8 15639683

[18] Higgins JPT, Altman DG, Gøtzsche PC, et al The Cochrane collaboration's tool for assessing risk of bias in randomised trials. BMJ 2011;343:d5928 10.1136/bmj.d5928 22008217 PMC3196245

[19] Higgins JP, Green S Cochrane Handbook for systematic reviews of interventions. John Wiley & Sons, 2011.

[20] Cioffi I, Farella M Quality of randomised controlled trials in dentistry. Int Dent J 2011;61:37–42. 10.1111/j.1875-595X.2011.00007.x 21382032 PMC9374841

[21] Djulbegovic B, Guyatt GH Progress in evidence-based medicine: a quarter century on. The Lancet 2017;390:415–23. 10.1016/S0140-6736(16)31592-6 28215660

[22] Moher D, Jadad AR, Nichol G, et al Assessing the quality of randomized controlled trials: an annotated bibliography of scales and checklists. Control Clin Trials 1995;16:62–73. 10.1016/0197-2456(94)00031-W 7743790

[23] Olivo SA, Macedo LG, Gadotti IC, et al Scales to assess the quality of randomized controlled trials: a systematic review. Phys Ther 2008;88:156–75. 10.2522/ptj.20070147 18073267

[24] Zeng X, Zhang Y, Kwong JSW, et al The methodological quality assessment tools for preclinical and clinical studies, systematic review and meta-analysis, and clinical practice guideline: a systematic review. J Evid Based Med 2015;8:2–10. 10.1111/jebm.12141 25594108

[25] Verhagen AP, de Vet HCW, de Bie RA, et al The art of quality assessment of RCTs included in systematic reviews. J Clin Epidemiol 2001;54:651–4. 10.1016/S0895-4356(00)00360-7 11438404

[26] Page MJ, McKenzie JE, Higgins JPT Tools for assessing risk of reporting biases in studies and syntheses of studies: a systematic review. BMJ Open 2018;8:e019703 10.1136/bmjopen-2017-019703 PMC5857645 29540417

[27] Sterne JAC, Savović J, Page MJ, et al Rob 2: a revised tool for assessing risk of bias in randomised trials. BMJ. In Press 2019;366:l4898 10.1136/bmj.l4898 31462531

[28] Moher D, Hopewell S, Schulz KF Consort 2010 statement: updated guidelines for reporting parallel group randomised trials. BMJ (Clinical research 2010;340. 10.1136/bmj.c332 PMC2844940 20332509

[29] Moher D, Shamseer L, Clarke M, et al Preferred reporting items for systematic review and meta-analysis protocols (PRISMA-P) 2015 statement. Syst Rev 2015;4:1 10.1186/2046-4053-4-1 25554246 PMC4320440

[30] Shamseer L, Moher D, Clarke M, et al Preferred reporting items for systematic review and meta-analysis protocols (PRISMA-P) 2015: elaboration and explanation. BMJ 2015;349:g7647 10.1136/bmj.g7647 25555855

[31] Moher D, Stewart L, Shekelle P Implementing PRISMA-P: recommendations for prospective authors: Biomed central 2016. 10.1186/s13643-016-0191-y PMC4730599 26822481

[32] Mills EJ, Chan A-W, Wu P, et al Design, analysis, and presentation of crossover trials. Trials 2009;10:27 10.1186/1745-6215-10-27 19405975 PMC2683810

[33] Sibbald B, Roberts C Understanding controlled trials: crossover trials. BMJ 1998;316:1719–20. 10.1136/bmj.316.7146.1719 9614025 PMC1113275

[34] Glanville JM, Lefebvre C, Miles JNV, et al How to identify randomized controlled trials in MEDLINE: ten years on. J Med Libr Assoc 2006;94. PMC1435857 16636704

[35] BMJ Publishing Group Limited Study design search filters. BMJ Best Practice 2019.

[36] Young A, Brookes S, Rumsey N, et al Agreement on what to measure in randomised controlled trials in burn care: study protocol for the development of a core outcome set. BMJ Open 2017;7:e017267 10.1136/bmjopen-2017-017267 PMC5734442 28669969

[37] Gates A, Gates M, Duarte G, et al Evaluation of the reliability, usability, and applicability of AMSTAR, AMSTAR 2, and ROBIS: protocol for a descriptive analytic study. Syst Rev 2018;7:85 10.1186/s13643-018-0746-1 29898777 PMC6000957

[38] Higgins J, Sterne J, Savović J, et al A revised tool for assessing risk of bias in randomized trials in: Chandler J, McKenzie J, Boutron I, Welch V (editors). Cochrane methods. Cochrane database of systematic reviews 2016;10.

[39] Armijo-Olivo S, Ospina M, da Costa BR, et al Poor reliability between Cochrane reviewers and blinded external reviewers when applying the Cochrane risk of bias tool in physical therapy trials. PLoS One 2014;9:e96920 10.1371/journal.pone.0096920 24824199 PMC4019638

[40] Graham N, Haines T, Goldsmith CH, et al Reliability of 3 assessment tools used to evaluate randomized controlled trials for treatment of neck pain. Spine 2012;37:515–22. 10.1097/BRS.0b013e31822671eb 21673624

[41] Hartling L, Hamm MP, Milne A, et al Testing the risk of bias tool showed low reliability between individual reviewers and across consensus assessments of reviewer pairs. J Clin Epidemiol 2013;66:973–81. 10.1016/j.jclinepi.2012.07.005 22981249

[42] Hartling L, Ospina M, Liang Y, et al Risk of bias versus quality assessment of randomised controlled trials: cross sectional study. BMJ 2009;339:b4012 10.1136/bmj.b4012 19841007 PMC2764034

[43] Bilandzic A, Fitzpatrick T, Rosella L, et al Risk of bias in systematic reviews of non-randomized studies of adverse cardiovascular effects of thiazolidinediones and cyclooxygenase-2 inhibitors: application of a new Cochrane risk of bias tool. PLoS Med 2016;13:e1001987 10.1371/journal.pmed.1001987 27046153 PMC4821619

[44] Hallgren KA Computing inter-rater reliability for observational data: an overview and tutorial. Tutor Quant Methods Psychol 2012;8:23–34. 10.20982/tqmp.08.1.p023 22833776 PMC3402032

[45] Nelson KP, Edwards D A measure of association for ordered categorical data in population-based studies. Stat Methods Med Res 2018;27:812–31. 10.1177/0962280216643347 27184590 PMC5112161

[46] Schulz KF, Altman DG, Moher D Consort 2010 statement: updated guidelines for reporting parallel group randomised trials. BMC Med 2010;8:18 10.1186/1741-7015-8-18 20334633 PMC2860339

[47] Horsley T, Galipeau J, Petkovic J, et al Reporting quality and risk of bias in randomised trials in health professions education. Med Educ 2017;51:61–71. 10.1111/medu.13130 27981660

