# Supplementary material for: Risk of bias and reporting completeness of randomised controlled trials in burn care: protocol for a systematic review
Source: BMJ Open. 2019 Dec 18;9(12):e033472. doi: 10.1136/bmjopen-2019-033472 (PMC6937119; doi:10.1136/bmjopen-2019-033472)
Supplement: Supplementary data [file bmjopen-2019-033472supp001.pdf]

**Assessors and assigned RCTs**

| RCT | A | B | C | D | E | F | n | number of RCTs: | 30 | #1-30 |
|---|---|---|---|---|---|---|---|---|---|---|
| 1 | yes | yes | yes | | | | 3 | ‘n’ assessors/RCT: | 3 | |
| 2 | yes | yes | | yes | | | 3 | ‘n’ assessments: | 90 | |
| 3 | yes | yes | | | yes | | 3 | ‘n’ assessors: | 6 | A-F |
| 4 | yes | yes | | | | yes | 3 | ‘n’ RCTs/assessor: | 15 | |
| 5 | yes | yes | yes | | | | 3 | | | |
| 6 | yes | yes | | yes | | | 3 | | | |
| 7 | yes | | yes | | yes | | 3 | | AB | 6 |
| 8 | yes | | | yes | | yes | 3 | | AC | 6 |
| 9 | yes | | yes | | yes | | 3 | | AD | 6 |
| 10 | yes | | | yes | | yes | 3 | | AE | 6 |
| 11 | yes | | | | yes | yes | 3 | | AF | 6 |
| 12 | yes | | | | yes | yes | 3 | | BC | 6 |
| 13 | yes | | yes | yes | | | 3 | | BD | 6 |
| 14 | yes | | yes | | | yes | 3 | | BE | 6 |
| 15 | yes | | | yes | yes | | 3 | | BF | 6 |
| 16 | | yes | | yes | | yes | 3 | | CD | 6 |
| 17 | | yes | yes | | yes | | 3 | | CE | 6 |
| 18 | | yes | | yes | yes | | 3 | | CF | 6 |
| 19 | | yes | | yes | yes | | 3 | | DE | 6 |
| 20 | | yes | | | yes | yes | 3 | | DF | 6 |
| 21 | | yes | yes | | | yes | 3 | | EF | 6 |
| 22 | | yes | yes | | | yes | 3 | | | 90 |
| 23 | | | yes | yes | yes | | 3 | | | |
| 24 | | yes | | | yes | yes | 3 | | | |
| 25 | | yes | yes | yes | | | 3 | | | |
| 26 | | | | yes | yes | yes | 3 | | | |
| 27 | | | yes | yes | | yes | 3 | | | |
| 28 | | | yes | yes | yes | | 3 | | | |
| 29 | | | yes | | yes | yes | 3 | | | |
| 30 | | | yes | yes | | yes | 3 | | | |
| | **15** | **15** | **15** | **15** | **15** | **15** | | | | |

**RCTs assessed by all pairs of assessors**

| RCT | AB | AC | AD | AE | AF | BC | BD | BE | BF | CD | CE | CF | DE | DF | EF |
|---|---|---|---|---|---|---|---|---|---|---|---|---|---|---|---|
| 1 | 1 | 1 | 0 | 0 | 0 | 1 | 0 | 0 | 0 | 0 | 0 | 0 | 0 | 0 | 0 |
| 2 | 1 | 0 | 1 | 0 | 0 | 0 | 1 | 0 | 0 | 0 | 0 | 0 | 0 | 0 | 0 |
| 3 | 1 | 0 | 0 | 1 | 0 | 0 | 0 | 1 | 0 | 0 | 0 | 0 | 0 | 0 | 0 |
| 4 | 1 | 0 | 0 | 0 | 1 | 0 | 0 | 0 | 1 | 0 | 0 | 0 | 0 | 0 | 0 |
| 5 | 1 | 1 | 0 | 0 | 0 | 1 | 0 | 0 | 0 | 0 | 0 | 0 | 0 | 0 | 0 |
| 6 | 1 | 0 | 1 | 0 | 0 | 0 | 1 | 0 | 0 | 0 | 0 | 0 | 0 | 0 | 0 |
| 7 | 0 | 1 | 0 | 1 | 0 | 0 | 0 | 0 | 0 | 0 | 1 | 0 | 0 | 0 | 0 |
| 8 | 0 | 0 | 1 | 0 | 1 | 0 | 0 | 0 | 0 | 0 | 0 | 0 | 0 | 1 | 0 |
| 9 | 0 | 1 | 0 | 1 | 0 | 0 | 0 | 0 | 0 | 0 | 1 | 0 | 0 | 0 | 0 |
| 10 | 0 | 0 | 1 | 0 | 1 | 0 | 0 | 0 | 0 | 0 | 0 | 0 | 0 | 1 | 0 |
| 11 | 0 | 0 | 0 | 1 | 1 | 0 | 0 | 0 | 0 | 0 | 0 | 0 | 0 | 0 | 1 |
| 12 | 0 | 0 | 0 | 1 | 1 | 0 | 0 | 0 | 0 | 0 | 0 | 0 | 0 | 0 | 1 |
| 13 | 0 | 1 | 1 | 0 | 0 | 0 | 0 | 0 | 0 | 1 | 0 | 0 | 0 | 0 | 0 |
| 14 | 0 | 1 | 0 | 0 | 1 | 0 | 0 | 0 | 0 | 0 | 0 | 1 | 0 | 0 | 0 |
| 15 | 0 | 0 | 1 | 1 | 0 | 0 | 0 | 0 | 0 | 0 | 0 | 0 | 1 | 0 | 0 |
| 16 | 0 | 0 | 0 | 0 | 0 | 0 | 1 | 0 | 1 | 0 | 0 | 0 | 0 | 1 | 0 |
| 17 | 0 | 0 | 0 | 0 | 0 | 1 | 0 | 1 | 0 | 0 | 1 | 0 | 0 | 0 | 0 |
| 18 | 0 | 0 | 0 | 0 | 0 | 0 | 1 | 1 | 0 | 0 | 0 | 0 | 1 | 0 | 0 |
| 19 | 0 | 0 | 0 | 0 | 0 | 0 | 1 | 1 | 0 | 0 | 0 | 0 | 1 | 0 | 0 |
| 20 | 0 | 0 | 0 | 0 | 0 | 0 | 0 | 1 | 1 | 0 | 0 | 0 | 0 | 0 | 1 |
| 21 | 0 | 0 | 0 | 0 | 0 | 1 | 0 | 0 | 1 | 0 | 0 | 1 | 0 | 0 | 0 |
| 22 | 0 | 0 | 0 | 0 | 0 | 1 | 0 | 0 | 1 | 0 | 0 | 1 | 0 | 0 | 0 |
| 23 | 0 | 0 | 0 | 0 | 0 | 0 | 0 | 0 | 0 | 1 | 1 | 0 | 1 | 0 | 0 |
| 24 | 0 | 0 | 0 | 0 | 0 | 0 | 0 | 1 | 1 | 0 | 0 | 0 | 0 | 0 | 1 |
| 25 | 0 | 0 | 0 | 0 | 0 | 1 | 1 | 0 | 0 | 1 | 0 | 0 | 0 | 0 | 0 |
| 26 | 0 | 0 | 0 | 0 | 0 | 0 | 0 | 0 | 0 | 0 | 0 | 0 | 1 | 1 | 1 |
| 27 | 0 | 0 | 0 | 0 | 0 | 0 | 0 | 0 | 0 | 1 | 0 | 1 | 0 | 1 | 0 |
| 28 | 0 | 0 | 0 | 0 | 0 | 0 | 0 | 0 | 0 | 1 | 1 | 0 | 1 | 0 | 0 |
| 29 | 0 | 0 | 0 | 0 | 0 | 0 | 0 | 0 | 0 | 0 | 1 | 1 | 0 | 0 | 1 |
| 30 | 0 | 0 | 0 | 0 | 0 | 0 | 0 | 0 | 0 | 1 | 0 | 1 | 0 | 1 | 0 |
| | **6** | **6** | **6** | **6** | **6** | **6** | **6** | **6** | **6** | **6** | **6** | **6** | **6** | **6** | **6** |
