# Supplementary material for: Risk of bias and reporting completeness of randomised controlled trials in burn care: protocol for a systematic review
Source: BMJ Open. 2019 Dec 18;9(12):e033472. doi: 10.1136/bmjopen-2019-033472 (PMC6937119; doi:10.1136/bmjopen-2019-033472)
Supplement: Author's manuscript [file bmjopen-2019-033472.draft_revisions.pdf]

BMJ Open is committed to open peer review. As part of this commitment we make the peer review history of every article we publish publicly available.

When an article is published we post the peer reviewers' comments and the authors' responses online. We also post the versions of the paper that were used during peer review. These are the versions that the peer review comments apply to.

The versions of the paper that follow are the versions that were submitted during the peer review process. They are not the versions of record or the final published versions. They should not be cited or distributed as the published version of this manuscript.

BMJ Open is an open access journal and the full, final, typeset and author-corrected version of record of the manuscript is available on our site with no access controls, subscription charges or pay-per-view fees (http://bmjopen.bmj.com).

If you have any questions on BMJ Open's open peer review process please email info.bmjopen@bmj.com

BMJ Open

Risk of bias and reporting completeness of randomised controlled trials in burn care: Protocol for a systematic review.

Journal:	*BMJ Open*
Manuscript ID	bmjopen-2019-033472
Article Type:	Protocol
Date Submitted by the Author:	06-Aug-2019
Complete List of Authors:	Young, Amber; University of Bristol, Bristol Centre for Surgical Research, Population Health Sciences, Bristol Medical School; University Hospitals Bristol NHS Foundation Trust, Paediatric Anaesthesia<br>Reeves, Barnaby; University of Bristol, School of clinical sciences<br>Cheng, Hung-Yuan; University of Bristol , Bristol Medical School<br>Wasiak, Jason; The University of Melbourne Faculty of Medicine Dentistry and Health Sciences<br>Muir, Duncan; University of Bristol Medical School<br>Davies, Anna; University of Bristol, Centre for Academic Child Health<br>Blazeby, Jane; University of Bristol, Bristol Centre for Surgical Research, Bristol Medical School
Keywords:	Burns, Randomised Controlled Trials as topic, Bias, Quality, CONSORT, Cochrane

SCHOLARONE™
Manuscripts

Risk of bias and reporting completeness of randomised controlled trials in burn care: Protocol for a systematic review.

Amber E Young*[1], Barnaby Reeves[2], Hung-Yuan Cheng[1], Jason Wasiak[3], Duncan Muir[1], Anna Davies[1], Jane M. Blazeby[1,4]

[1]University of Bristol, Bristol Centre for Surgical Research, Bristol Medical School, Canynge Hall, 39 Whatley Road, Bristol UK, BS8 2PS, UK.

[2] University of Bristol, School of Clinical Sciences, Level 7, Bristol Royal Infirmary, Marlborough St, Bristol, UK, BS2 8HW, UK.

[3] Faculty of Medicine, Dentistry and Health Sciences, Melbourne School of Health Sciences, The University of Melbourne, Victoria, Australia.

[4] NIHR Biomedical Research Centre at University Hospitals Bristol NHS Foundation Trust and the University of Bristol UK.

*Corresponding author: Dr Amber E Young

Tel: 0117 3427910 email: amber.young1@nhs.net

Word count: 1,813

For peer review only

Abstract

Introduction: Burn care represents a healthcare and economic burden to patients internationally. Choice of the most clinically effective treatment strategies requires evidence which is best obtained through high quality randomised controlled trials (RCT). The number of published RCTs of burn care is increasing. However, trial quality and reporting standards are unclear. This study will assess the risk of bias and adequacy of reporting in recent burn care RCTs using tools endorsed by the Cochrane Collaboration.

Methods and analysis: A systematic literature review will be undertaken, assessing parallel group RCTs evaluating therapeutic interventions for patients with cutaneous burns. Literature searches will use Ovid MEDLINE, Ovid EMBASE, Web of Science and the Cochrane Library. Separate searches for each database will include medical subject heading and free text terms including 'burn', 'scald' 'thermal injury' and 'RCT'. Two reviewers will independently assess each study for inclusion. Risk of bias (RoB) will be assessed with the revised tool (RoB 2) and reporting completeness with the CONsolidated Standards of Reporting Trials (CONSORT 2010) guidelines. We will report a narrative synthesis of all

For peer review only - http://bmjopen.bmj.com/site/about/guidelines.xhtml

studies, including domain-specific, and overall risk of bias for the primary outcome of each trial. Inter-rater agreement for RoB 2 will be reported using Fleiss's Kappa. For adherence to the CONSORT guidelines, we will generate a completeness of reporting index for the five domains.

Ethics and dissemination: No ethics approval is required because published documents will be used. Findings of the study will be disseminated in a peer-reviewed journal and presented at conferences.

PROSPERO registration: CRD42018111020

Keywords

Burns; Randomised Controlled Trials as topic; Bias; quality; CONSORT; Cochrane

Strengths and Limitations:

- This systematic review will be the first to assess both the internal validity of burn care randomised controlled trials using the Risk of Bias 2 (RoB 2) tool and adherence to CONsolidated Standards of Reporting Trials (CONSORT 2010) reporting guidelines.
- The inter-rater reliability of RoB 2 will be assessed using six reviewers from different research backgrounds to simulate real world conditions.
- The systematic review will be limited to five-years and to articles published in English.
- Assessments of external validity will not be performed.

1. Introduction

For peer review only - http://bmjopen.bmj.com/site/about/guidelines.xhtml

In 2009, Chalmers and colleagues estimated clinical research waste to be 85% of global investment[1]. Wastage was attributed to methodological flaws in randomised controlled trials (RCTs). Poor quality RCTs may result in misleading conclusions increasing research waste [2-4]. Numbers of RCTS in burn care research are increasing, as new technologies are regularly introduced [5-9]. However, trial quality and adherence to reporting standards are uncertain[10-13]. Previous studies are limited by their lack of comprehensiveness and methods of quality assessment[14-16]. Quality assessment of RCTs involves evaluating internal and external validity. External validity assesses whether study results can be generalised to other populations[17]. Internal validity, or risk of bias (ROB), is the extent to which the study design is free from bias; a systematic error that leads to a deviation of results from the truth[18]. Transparent reporting is necessary to allow a clear assessment of the trial design and conduct as recommended by the Cochrane Collaboration [19 20].

Using an objective method to assess RoB and reporting completeness has become increasingly common and considered good practice [18 21]. For assessment of RoB, many tools are available, including overall scores and checklists[22 23]. The Cochrane collaboration endorse a different type of tool based on individual domains[18]. RoB domains are assessed separately, with the overall RoB is formed relating to the highest domain score. Accumulating evidence shows that this is the best tool for assessing the ROB of RCTs[24-26]. A revised version of the tool (RoB 2) has been developed which now examines single trial outcomes, includes 'signalling questions' to aid decision-making and a method to allow an overall RoB judgement [27]. As yet, this tool has not been tested for inter-rater reliability. The Cochrane collaboration recommend 'The CONsolidated Standards of Reporting Trials (CONSORT) statement' to assess adherence to reporting standards[28].

For peer review only - http://bmjopen.bmj.com/site/about/guidelines.xhtml

The aim of this review is to assess the risk of bias and adherence to reporting standards using Cochrane-approved tools. It will also assess inter-rater reliability of the new RoB 2 tool.

2. Methods

This review will meet its aim with these objectives, it will:

- Determine the number of parallel-group, individually randomised trials assessing burn care interventions published over the last five years and retrieve included full text articles.
- Assess the internal validity of the included RCTs using the revised Cochrane-endorsed Risk of Bias (RoB 2) tool.
- Assess the inter-rater reliability for the new RoB 2 tool.
- Assess the adherence of the included RCTs to CONsolidated Standards of Reporting Trials (CONSORT 2010) reporting guidelines.

b. Literature review search strategy

The systematic review will adhere to this pre-specified protocol and the Preferred Reporting Items for Systematic Reviews and Meta-Analyses (PRISMA) statement. This protocol will be aligned to the PRISMA-P statement[29-31]. It has been registered with the PROSPERO international prospective register of systematic reviews (CRD42018111020). We will report any amendments to the protocol that occur while undertaking the study, within the final manuscript.

I. Study Eligibility

For peer review only - http://bmjopen.bmj.com/site/about/guidelines.xhtml

Types of studies: Included studies must be full text individually randomised parallel RCTs published in peer-reviewed journals limited to those allocating human subjects to an intervention or control group within over the previous five years. We will exclude RCT protocols, conference proceedings, abstracts, non-English language publications, interim analysis reports, and studies not involving human subjects. Health economic evaluation reports of clinical trials will be considered if they contain enough information for assessing risk of bias (e.g. clearly described methods for the trial conduct). We will also exclude trials that compare treatments *within* subjects as there is not yet a RoB 2 tool designed for assessing such trials. Also, in burn care research, these are typically not cross-over trials as generally defined[32 33]. Instead, they commonly use two wounds or two parts of the same wound. It is uncertain what the dependence between these might be, or how treatments might "contaminate" one another.

Types of participants: We will include studies evaluating two or more interventions in patients of any age with cutaneous burns. Studies where the population consists of patients with combined burn and mechanical injuries will be excluded, as the data relating to burn patients alone is likely to be difficult to disaggregate.

Type of interventions: Interventions to treat cutaneous burns of any aetiology.

Types of outcome: Clinical or patient-reported outcomes. Laboratory studies will be excluded.

II. Identification of studies

For peer review only - http://bmjopen.bmj.com/site/about/guidelines.xhtml

A pre-defined search strategy will be designed by the authors in conjunction with experienced systematic reviewers to identify RCTs in the field of burn care. Electronic searches of Ovid MEDLINE, Ovid EMBASE, Web of Science and The Cochrane Library will be searched using medical subject heading and free text terms including 'burn', 'scald' 'thermal injury' and 'RCT'. To limit the search to RCTs, we will use terms derived from published RCT search strategies on Medline and the BMJ best practice guideline[34]. The thesaurus vocabulary of each database will be used to adapt the search terms. The search strategy for Ovid MEDLINE is included in a previous publication and in Table 1[35].

Ovid MEDLINE search strategy
1. Burns/ (MESH) exp
2. Burn*.tw
3. Scald*.tw
4. Thermal* adj injur*
5. 1 OR 2 OR 3 OR 4
6. Heartburn
7. Burnout
8. Burn* adj out
9. Burning
10. Burnetii
11. Burnish*
12. Burnet*
13. 6 OR 7 OR 8 OR 9 OR 10 OR 11 OR 12
14. 5 NOT 13
15. Limit to RCT, clinical trial, English language, human, last five years

Table 1: Ovid MEDLINE search strategy

III. Study selection process:

Before screening abstracts and full text papers, authors undertaking study selection, RoB 2 assessment and data extraction, will undergo training to ensure a comparable

For peer review only - http://bmjopen.bmj.com/site/about/guidelines.xhtml

understanding of the purpose of the review and the eligibility criteria. The reference management software EndNote (Endnote X8 Clarivate Analytics) will be used to compile all titles derived from the initial searches, with duplicates removed, for the review authors to screen titles and abstracts against the eligibility criteria. Screening of titles and abstracts will be completed by one reviewer. Of these, 20% will be checked by another reviewer independently (AY, DM). Any studies appearing to meet the inclusion criteria based on the abstract will be retrieved as full-text articles. Two reviewers will then read the full-text articles in their entirety to assess for eligibility, with decisions on inclusion and exclusion recorded. Reasons for exclusion will be ordered hierarchically from most to least important (Table 2) and applied to each full text paper. The most important reason for exclusion met by a paper will be recorded as the reason for exclusion. Any disagreements will be discussed with senior researchers (JMB, BR).

Duplicate
Not published within time period
Population not consisting of burn patients alone
Not a parallel group RCT
Not in English
Non-human/animal study
No full text available
Laboratory based study
Volunteer study
Oesophageal burns only
Ocular burns only
Anaesthetic/sedation technique only (pain management included)
Smoke inhalation injuries without an associated cutaneous burn
Diagnostic test trial

For peer review only

Protocol only

Table 2: Reasons for full text exclusion

d. Data extraction and analysis:

All studies will be assessed by two reviewers independently and then in duplicate (AY, DM), with disagreements resolved by discussion until consensus is reached, or by a senior reviewer (BR). Data will be extracted into a specifically designed data extraction Microsoft Excel spreadsheet which will be specifically designed for this study. It will be adapted from the RoB 2 tool to include details for the CONSORT checklist by one of the co-authors (VC). Before starting the assessment, reviewers will undergo training with the spreadsheet. The reviewers will then independently pilot test each tool on five RCTs. If there are significant differences in the application of the tools in the pilot round, additional testing will be undertaken[36].

Data extracted will include study details and research design. Study details will include author, publication year, number of sites and number of participants recruited per trial, design (full RCT, pilot study) and intervention tested. Risk of bias judgement will be reported for all trial primary outcomes at a domain and overall level. Completeness of reporting will be reported for all RCTs.

I. Assessment of Risk of Bias

For peer review only - http://bmjopen.bmj.com/site/about/guidelines.xhtml

Included studies will be objectively assessed for internal validity using the revised risk of bias tool (RoB 2)[37]. The tool has five domains to assess bias arising from randomisation, deviations from intended interventions, missing outcome data, outcome measurement and in selection of the reported result. The assessment is specific to a single trial outcome. Categories of the overall risk of bias for the study outcome are low (risk of bias is low for all domains), some concerns (some concerns in at least one domain) and high (high risk of bias for at least one domain or some concerns for multiple domains).

Choice of outcome: Risk of bias will be assessed for the treatment effect for the primary outcome in each included trial. If this is not reported explicitly, we will use the following decision rule to select the treatment effect to report: we will assess the treatment effect for the outcome used to calculate sample size and, if this too is not reported, assess the treatment effect for the outcome named in the title, then the first reported outcome in the results[36].

II. Assessment of the inter-rater agreement of the RoB 2 tool [38 39]

As the revised risk of bias tool (RoB 2) is new, measuring agreement between reviewers will help assess whether the new guidance with signalling questions has improved the reliability of the tool compared to the previous version[40-42]. To evaluate the inter-rater agreement, six independent reviewers will assess the same 30 studies allocated in a balanced incomplete blocks design (please see Appendix A), ensuring that each study is rated three times, each assessor rates 15 studies and all pairs of assessors rate six studies. Inter-rater agreement will be measured for each domain of bias, and for the overall RoB judgment, by calculating Fleiss's Kappa scores[43 44]. We will categorise

For peer review only - http://bmjopen.bmj.com/site/about/guidelines.xhtml

agreement as poor (0.00), slight (0.01-0.20), fair (0.21-0.40), moderate (0.41-0.60), substantial (0.61-0.80), or almost perfect (0.81-1.00)[26].

III. Assessment of reporting completeness

Completeness of reporting for each included RCT will be assessed using the latest revision of the CONSORT statement checklist[45]. For assessing completeness of reporting, we will calculate a reporting index defined as the percentage of items reported in each of five domains (title/abstract, introduction, methods, results and discussion), as has been done previously[46].

Patient and Public Involvement: There was no patient or public involvement in designing the study or writing up the study protocol.

Ethical issues

This study involves analysis of data from published literature and does not involve individual-level identifiable data. Given this, there were no privacy concerns that required ethical approval. Results from this study will be presented at conferences and through publication in a peer-reviewed journal.

Dissemination: we will publish the findings of this study in a peer-reviewed journal accessible to systematic review and burn care researchers.

Funding: This work was supported by a National Institute for Health Research Doctoral Research Fellowship DRF-2016-09-031. The views expressed are those of the author(s) and

For peer review only - http://bmjopen.bmj.com/site/about/guidelines.xhtml

not necessarily those of the NHS, the NIHR or the Department of Health and Social Care. JMB is part-funded by the Bristol Biomedical Research Centre at the University Hospitals Bristol NHS Foundation Trust and the University of Bristol; Grant no. BRC-1215-2001. JMB is an NIHR Senior Investigator.

Disclaimer: The views expressed are those of the author(s) and not necessarily those of the NHS, the NIHR or the Department of Health.

Author statement: Contributors AEY wrote the paper and conceived the project with the support of JMB and BR. H-YC devised the interactive study-specific data extraction spreadsheet. DM contributed to data extraction. JMB, BR, AD and H-YC edited and critically revised the article. All authors have read and approved the manuscript. AEY is the guarantor of the review.

Competing interests: None declared

I, the Submitting Author has the right to grant and does grant on behalf of all authors of the Work (as defined in the below author licence), an exclusive licence and/or a non-exclusive licence for contributions from authors who are: i) UK Crown employees; ii) where BMJ has agreed a CC-BY licence shall apply, and/or iii) in accordance with the terms applicable for US Federal Government officers or employees acting as part of their official duties; on a worldwide, perpetual, irrevocable, royalty-free basis to BMJ Publishing Group Ltd ("BMJ") its licensees and where the relevant Journal is co-owned by BMJ to the co-owners of the Journal, to publish the Work in BMJ Open and any other BMJ products and to exploit all rights, as set out in our licence.

The Submitting Author accepts and understands that any supply made under these terms is made by BMJ to the Submitting Author unless you are acting as an employee on behalf of your employer or a postgraduate student of an affiliated institution which is paying any

For peer review only

applicable article publishing charge ("APC") for Open Access articles. Where the Submitting Author wishes to make the Work available on an Open Access basis (and intends to pay the relevant APC), the terms of reuse of such Open Access shall be governed by a Creative Commons licence – details of these licences and which Creative Commons licence will apply to this Work are set out in our licence referred to above.

References:

1. Chalmers I, Glasziou P. Avoidable waste in the production and reporting of research evidence. *The Lancet* 2009;374(9683):86-89.
2. Yordanov Y, Dechartres A, Porcher R, et al. Avoidable waste of research related to inadequate methods in clinical trials. *BMJ* 2015;350:h809.
3. Ioannidis JP, Greenland S, Hlatky MA, et al. Increasing value and reducing waste in research design, conduct, and analysis. *The Lancet* 2014;383(9912):166-75.
4. Jüni P, Altman DG, Egger M. Assessing the quality of controlled clinical trials. *BMJ* 2001;323(7303):42-46.
5. Jeschke MG, Finnerty CC, Shahrokhi S, et al. Wound coverage technologies in burn care: novel techniques. *Journal of Burn Care & Research* 2013;34(6):612-20.
6. Selig HF, Lumenta DB, Giretzlehner M, et al. The properties of an "ideal" burn wound dressing–what do we need in daily clinical practice? Results of a worldwide online survey among burn care specialists. *Burns* 2012;38(7):960-66.
7. Jeschke MG, Herndon DN. Burns in children: standard and new treatments. *The Lancet* 2014;383(9923):1168-78. doi: 10.1016/s0140-6736(13)61093-4
8. Shahrokhi S, Arno A, Jeschke MG. The use of dermal substitutes in burn surgery: acute phase. *Wound Repair and Regeneration* 2014;22(1):14-22.
9. Young AE, Davies A, Bland S, et al. Systematic review of clinical outcome reporting in randomised controlled trials of burn care. *BMJ open* 2019;9(2):e025135.
10. Norman G, Christie J, Liu Z, et al. Antiseptics for burns. *Cochrane Database of Systematic Reviews* 2017(7)
11. Breederveld RS, Tuinebreijer WE. Recombinant human growth hormone for treating burns and donor sites. *Cochrane Database Syst Rev* 2012;12
12. Barajas-Nava LA, Lopez-Alcalde J, Roqué i Figuls M, et al. Antibiotic prophylaxis for preventing burn wound infection. *Cochrane Database of Systematic Reviews* 2013;6
13. Wasiak J, Cleland H, Campbell F, et al. Dressings for superficial and partial thickness burns. *The Cochrane Library* 2013
14. Danilla S, Wasiak J, Searle S, et al. Methodological quality of randomised controlled trials in burns care. A systematic review. *Burns* 2009;35(7):956-61.
15. Al-Benna S, Alzoubaidi D, Al-Ajam Y. Evidence-based burn care—an assessment of the methodological quality of research published in burn care journals from 1982 to 2008. *Burns* 2010;36(8):1190-95.
16. Sinha S, Yoon G, Shin W, et al. Burn clinical trials: A systematic review of registration and publications. *Burns* 2018;44(2):263-71.
17. Rothwell PM. External validity of randomised controlled trials:"to whom do the results of this trial apply?". *The Lancet* 2005;365(9453):82-93.
18. Higgins JP, Altman DG, Gøtzsche PC, et al. The Cochrane Collaboration's tool for assessing risk of bias in randomised trials. *BMJ* 2011;343:d5928.

For peer review only - http://bmjopen.bmj.com/site/about/guidelines.xhtml

19. Higgins JP, Green S. Cochrane handbook for systematic reviews of interventions: John Wiley & Sons 2011.
20. Cioffi I, Farella M. Quality of randomised controlled trials in dentistry. *International Dental Journal* 2011;61(1):37-42.
21. Djulbegovic B, Guyatt GH. Progress in evidence-based medicine: a quarter century on. *The Lancet* 2017;390(10092):415-23.
22. Moher D, Jadad AR, Nichol G, et al. Assessing the quality of randomized controlled trials: an annotated bibliography of scales and checklists. *Controlled Clinical Trials* 1995;16(1):62-73.
23. Olivo SA, Macedo LG, Gadotti IC, et al. Scales to assess the quality of randomized controlled trials: a systematic review. *Physical Therapy* 2008;88(2):156-75.
24. Zeng X, Zhang Y, Kwong JS, et al. The methodological quality assessment tools for preclinical and clinical studies, systematic review and meta-analysis, and clinical practice guideline: a systematic review. *Journal of Evidence-based Medicine* 2015;8(1):2-10.
25. Verhagen AP, de Vet HC, de Bie RA, et al. The art of quality assessment of RCTs included in systematic reviews. *Journal of Clinical Epidemiology* 2001;54(7):651-54.
26. Page MJ, McKenzie JE, Higgins JP. Tools for assessing risk of reporting biases in studies and syntheses of studies: a systematic review. *BMJ open* 2018;8(3):e019703.
27. al SJe. RoB 2: a revised tool for assessing risk of bias in randomised trials. *BMJ* 2019;(in press)
28. Moher D, Hopewell S, Schulz KF, et al. CONSORT 2010 statement: updated guidelines for reporting parallel group randomised trials. *BMJ (Clinical research ed)* 2010;340:c332.
29. Moher D, Shamseer L, Clarke M, et al. Preferred reporting items for systematic review and meta-analysis protocols (PRISMA-P) 2015 statement. *Systematic reviews* 2015;4(1):1.
30. Shamseer L, Moher D, Clarke M, et al. Preferred reporting items for systematic review and meta-analysis protocols (PRISMA-P) 2015: elaboration and explanation. *BMJ* 2015;349:g7647.
31. Moher D, Stewart L, Shekelle P. Implementing PRISMA-P: recommendations for prospective authors: BioMed Central, 2016.
32. Mills EJ, Chan A-W, Wu P, et al. Design, analysis, and presentation of crossover trials. *Trials* 2009;10(1):27.
33. Sibbald B, Roberts C. Understanding controlled trials Crossover trials. *BMJ* 1998;316(7146):1719-20.
34. Glanville JM, Lefebvre C, Miles JN, et al. How to identify randomized controlled trials in MEDLINE: ten years on. *Journal of the Medical Library Association* 2006;94(2):130.
35. Young AB, S.; Rumsey, N.; Blazeby, J. Agreement on what to measure in randomised controlled trials in burn care: study protocol for the development of a core outcome set. *BMJ Open* 2017;7(6) doi: 10.1136/bmjopen-2017-017267
36. Gates A, Gates M, Duarte G, et al. Evaluation of the reliability, usability, and applicability of AMSTAR, AMSTAR 2, and ROBIS: protocol for a descriptive analytic study. *Systematic Reviews* 2018;7(1):85.
37. Higgins J, Sterne J, Savović J, et al. A revised tool for assessing risk of bias in randomized trials In: Chandler J, McKenzie J, Boutron I, Welch V (editors). Cochrane Methods. *Cochrane Database of Systematic Reviews* 2016;10
38. Hartling L, Ospina M, Liang Y, et al. Risk of bias versus quality assessment of randomised controlled trials: cross sectional study. *BMJ* 2009;339:b4012.
39. Bilandzic A, Fitzpatrick T, Rosella L, et al. Risk of bias in systematic reviews of non-randomized studies of adverse cardiovascular effects of thiazolidinediones and cyclooxygenase-2 inhibitors: application of a new cochrane risk of bias tool. *PLoS Medicine* 2016;13(4):e1001987.
40. Armijo-Olivo S, Ospina M, da Costa BR, et al. Poor reliability between Cochrane reviewers and blinded external reviewers when applying the Cochrane risk of bias tool in physical therapy trials. *PloS One* 2014;9(5):e96920.

For peer review only - http://bmjopen.bmj.com/site/about/guidelines.xhtml

41. Graham N, Haines T, Goldsmith CH, et al. Reliability of 3 assessment tools used to evaluate randomized controlled trials for treatment of neck pain. *Spine* 2012;37(6):515-22.
42. Hartling L, Hamm MP, Milne A, et al. Testing the risk of bias tool showed low reliability between individual reviewers and across consensus assessments of reviewer pairs. *Journal of Clinical Epidemiology* 2013;66(9):973-81.
43. Hallgren KA. Computing inter-rater reliability for observational data: an overview and tutorial. *Tutorials in quantitative methods for psychology* 2012;8(1):23.
44. Nelson KP, Edwards D. A measure of association for ordered categorical data in population-based studies. *Statistical methods in medical research* 2018;27(3):812-31.
45. Schulz KF, Altman DG, Moher D. CONSORT 2010 statement: updated guidelines for reporting parallel group randomised trials. *BMC medicine* 2010;8(1):18.
46. Horsley T, Galipeau J, Petkovic J, et al. Reporting quality and risk of bias in randomised trials in health professions education. *Medical education* 2017;51(1):61-71.

For peer review only

For peer review only - http://bmjopen.bmj.com/site/about/guidelines.xhtml

Population Health Sciences
University of Bristol
Canynge Hall
Whatley Road
Bristol
BS8 2PN

August 6th 2019

Dear BMJ Open editorial team

I am pleased to submit the following original article**: Risk of bias and reporting completeness of randomised controlled trials in burn care: Protocol for a systematic review** for consideration for publication in the British Medical Journal Open.

The paper describes a protocol for a systematic review. This study will be the first to assess both the internal validity of burn care randomised controlled trials using the Risk of Bias 2 (RoB 2) tool and adherence to CONsolidated Standards of Reporting Trials (CONSORT 2010) reporting guidelines. The inter-rater reliability of the new RoB 2 tool will also be assessed using six reviewers from different research backgrounds to simulate real world conditions.

The research project has full ethical approval (REC reference: 17/SW/0025; IRAS project ID:221625), is funded by the NIHR through a Doctoral Research Fellowship DRF-2016-09-031. The paper has not been published before and is not under review for publication elsewhere.

Please let me know if you need any further information.

Thank you for your kind attention.

Best wishes

Dr Amber Young
Senior Research fellow
University of Bristol
Amber.young1@nhs.net
07771 547597

For peer review only

For peer review only - http://bmjopen.bmj.com/site/about/guidelines.xhtml

PRISMA-P (Preferred Reporting Items for Systematic review and Meta-Analysis Protocols) 2015 checklist: recommended items to address in a systematic review protocol*

Section and topic	Item No	Checklist item	
**ADMINISTRATIVE INFORMATION**			
Title:			
Identification	1a	Identify the report as a protocol of a systematic review	p1
Update	1b	If the protocol is for an update of a previous systematic review, identify as such	N/A
Registration	2	If registered, provide the name of the registry (such as PROSPERO) and registration number	p1
Authors:			
Contact	3a	Provide name, institutional affiliation, e-mail address of all protocol authors; provide physical mailing address of corresponding author	p1
Contributions	3b	Describe contributions of protocol authors and identify the guarantor of the review	p7
Amendments	4	If the protocol represents an amendment of a previously completed or published protocol, identify as such and list changes; otherwise, state plan for documenting important protocol amendments	p4
Support:			
Sources	5a	Indicate sources of financial or other support for the review	p7
Sponsor	5b	Provide name for the review funder and/or sponsor	p7
Role of sponsor or funder	5c	Describe roles of funder(s), sponsor(s), and/or institution(s), if any, in developing the protocol	
**INTRODUCTION**			
Rationale	6	Describe the rationale for the review in the context of what is already known	p2-3
Objectives	7	Provide an explicit statement of the question(s) the review will address with reference to participants, interventions, comparators, and outcomes (PICO)	p4
**METHODS**			
Eligibility criteria	8	Specify the study characteristics (such as PICO, study design, setting, time frame) and report characteristics (such as years considered, language, publication status) to be used as criteria for eligibility for the review	p4
Information sources	9	Describe all intended information sources (such as electronic databases, contact with study authors, trial registers or other grey literature sources) with planned dates of coverage	p5
Search strategy	10	Present draft of search strategy to be used for at least one electronic database, including planned limits, such that it could be repeated	Table 1
Study records:			
Data management	11a	Describe the mechanism(s) that will be used to manage records and data throughout the review	p5

For peer review only

Selection process	11b	State the process that will be used for selecting studies (such as two independent reviewers) through each phase of the review (that is, screening, eligibility and inclusion in meta-analysis)	p5
Data collection process	11c	Describe planned method of extracting data from reports (such as piloting forms, done independently, in duplicate), any processes for obtaining and confirming data from investigators	p5
Data items	12	List and define all variables for which data will be sought (such as PICO items, funding sources), any pre-planned data assumptions and simplifications	p5-6
Outcomes and prioritization	13	List and define all outcomes for which data will be sought, including prioritization of main and additional outcomes, with rationale	p5-6
Risk of bias in individual studies	14	Describe anticipated methods for assessing risk of bias of individual studies, including whether this will be done at the outcome or study level, or both; state how this information will be used in data synthesis	p6
Data synthesis	15a	Describe criteria under which study data will be quantitatively synthesised	p6
	15b	If data are appropriate for quantitative synthesis, describe planned summary measures, methods of handling data and methods of combining data from studies, including any planned exploration of consistency (such as I^2, Kendall's τ)	
	15c	Describe any proposed additional analyses (such as sensitivity or subgroup analyses, meta-regression)	
	15d	If quantitative synthesis is not appropriate, describe the type of summary planned	
Meta-bias(es)	16	Specify any planned assessment of meta-bias(es) (such as publication bias across studies, selective reporting within studies)	
Confidence in cumulative evidence	17	Describe how the strength of the body of evidence will be assessed (such as GRADE)	

*** It is strongly recommended that this checklist be read in conjunction with the PRISMA-P Explanation and Elaboration (cite when available) for important clarification on the items. Amendments to a review protocol should be tracked and dated.** **The copyright for PRISMA-P (including checklist) is held by the PRISMA-P Group and is distributed under a Creative Commons Attribution Licence 4.0.**

From: Shamseer L, Moher D, Clarke M, Ghersi D, Liberati A, Petticrew M, Shekelle P, Stewart L, PRISMA-P Group. Preferred reporting items for systematic review and meta-analysis protocols (PRISMA-P) 2015: elaboration and explanation. BMJ. 2015 Jan 2;349(jan02 1):g7647.

For peer review only

NHS
Health Research Authority

South West - Frenchay Research Ethics Committee
Level 3, Block B
Whitefriars
Lewins Mead,
Bristol BS1 2NT
Email: nrescommittee.southwest-frenchay@nhs.net
Tel: 0207 1048 045

<u>Please note: This is the favourable opinion of the REC only and does not allow</u> the amendment to be implemented at NHS sites in England until the outcome of the HRA assessment has been confirmed.

12 June 2019

Dr Amber E R Young
School of Social and Community Medicine
University of Bristol
Bristol
BS8 2PN

Dear Dr Young

**Study title:**	**Optimising evidence-based decision-making for patients withBurns by the development of a Core Outcome Set and theidentification of outcome measurement tools (COSB).**
**REC reference:**	**17/SW/0025**
**Protocol number:**	**2717**
**Amendment number:**	**2**
**Amendment date:**	**20 March 2019**
**IRAS project ID:**	**221625**

The above amendment was reviewed by the Sub-Committee in correspondence.

Ethical opinion

The members of the Committee taking part in the review gave a favourable ethical opinion of the amendment on the basis described in the notice of amendment form and supporting documentation.

The amendment related to changes to the protocol and documents required to reopen recruitment for the qualitative interviews in phase one of the study

Approved documents

The documents reviewed and approved at the meeting were:

Document	*Version*	*Date*
Covering letter on headed paper [Cover Letter]	N/A	12 March 2019
Interview schedules or topic guides for participants [Interview topic guide]	2.0	12 March 2019
Notice of Substantial Amendment (non-CTIMP) [Substantial Amendment Form]	2	20 March 2019

For peer review only

Other [Statement of Activities]	2.0	12 March 2019
Participant consent form [Consent form (Adult)]	2.0	12 March 2019
Participant consent form [Consent form (Parent of child)]	2.0	12 March 2019
Participant consent form [Consent form (Participant - Parent)]	2.0	12 March 2019
Participant consent form [Consent form (Staff)]	2.0	12 March 2019
Participant consent form [Consent form (Child 10-15 years)]	2.0	12 March 2019
Participant information sheet (PIS) [PIS (Participant - Parent)]	2.0	12 March 2019
Participant information sheet (PIS) [PIS (Adult)]	2.0	12 March 2019
Participant information sheet (PIS) [PIS (Child)]	2.0	12 March 2019
Participant information sheet (PIS) [PIS (Parent)]	2.0	12 March 2019
Participant information sheet (PIS) [Staff Information Sheet]	2.0	12 March 2019
Research protocol or project proposal [Protocol]	3.0	12 March 2019

Membership of the Committee

The members of the Committee who took part in the review are listed on the attached sheet.

Working with NHS Care Organisations

Sponsors should ensure that they notify the R&D office for the relevant NHS care organisation of this amendment in line with the terms detailed in the categorisation email issued by the lead nation for the study.

Statement of compliance

The Committee is constituted in accordance with the Governance Arrangements for Research Ethics Committees and complies fully with the Standard Operating Procedures for Research Ethics Committees in the UK.

HRA Learning

We are pleased to welcome researchers and research staff to our HRA Learning Events and online learning opportunities– see details at: https://www.hra.nhs.uk/planning-and-improving-research/learning/

**17/SW/0025:**	**Please quote this number on all correspondence**

Yours sincerely

Mr Stephen Draper
Chair

E-mail: nrescommittee.southwest-frenchay@nhs.net

Enclosures: *List of names and professions of members who took part in the review*

For peer review only

Health Research Authority

Copy to: *Dr Amber E R Young*

South West - Frenchay Research Ethics Committee

Attendance at Sub-Committee of the REC meeting on 19 May 2019

Committee Members:

Name	*Profession*	*Present*	*Notes*
Mr Stephen Draper	Retired Head teacher	Yes	
Dr Tarnjit Kaur Khera	Clinical Research Manager	Yes	

Also in attendance:

Name	*Position (or reason for attending)*
Miss Natasha Bridgeman	REC Manager

For peer review only

Assessors and assigned RCTs

RCT	A	B	C	D	E	F	n
1	yes	yes	yes				3
2	yes	yes		yes			3
3	yes	yes			yes		3
4	yes	yes				yes	3
5	yes	yes	yes				3
6	yes	yes		yes			3
7	yes		yes		yes		3
8	yes			yes		yes	3
9	yes		yes		yes		3
10	yes			yes		yes	3
11	yes				yes	yes	3
12	yes				yes	yes	3
13	yes		yes	yes			3
14	yes		yes			yes	3
15	yes			yes	yes		3
16		yes		yes		yes	3
17		yes	yes		yes		3
18		yes		yes	yes		3
19		yes		yes	yes		3
20		yes			yes	yes	3
21		yes	yes			yes	3
22		yes	yes			yes	3
23			yes	yes	yes		3
24		yes			yes	yes	3
25		yes	yes	yes			3
26				yes	yes	yes	3
27			yes	yes		yes	3
28			yes	yes	yes		3
29			yes		yes	yes	3
30			yes	yes		yes	3
	**15**	**15**	**15**	**15**	**15**	**15**	

**number of RCTs:**	**30**	**#1-30**
‘n’ assessors/RCT:	3	
‘n’ assessments:	90	
‘n’ assessors:	6	A-F
‘n’ RCTs/assessor:	15	
	AB	6
	AC	6
	AD	6
	AE	6
	AF	6
	BC	6
	BD	6
	BE	6
	BF	6
	CD	6
	CE	6
	CF	6
	DE	6
	DF	6
	EF	6
		90

RCTs assessed by all pairs of assessors

AB	AC	AD	AE	AF	BC	BD	BE	BF	CD	CE	CF	DE	DF	EF
1	1	0	0	0	1	0	0	0	0	0	0	0	0	0
1	0	1	0	0	0	1	0	0	0	0	0	0	0	0
1	0	0	1	0	0	0	1	0	0	0	0	0	0	0
1	0	0	0	1	0	0	0	1	0	0	0	0	0	0
1	1	0	0	0	1	0	0	0	0	0	0	0	0	0
1	0	1	0	0	0	1	0	0	0	0	0	0	0	0
0	1	0	1	0	0	0	0	0	0	1	0	0	0	0
0	0	1	0	1	0	0	0	0	0	0	0	0	1	0
0	1	0	1	0	0	0	0	0	0	1	0	0	0	0
0	0	1	0	1	0	0	0	0	0	0	0	0	1	0
0	0	0	1	1	0	0	0	0	0	0	0	0	0	1
0	0	0	1	1	0	0	0	0	0	0	0	0	0	1
0	1	1	0	0	0	0	0	0	1	0	0	0	0	0
0	1	0	0	1	0	0	0	0	0	0	1	0	0	0
0	0	1	1	0	0	0	0	0	0	0	0	1	0	0
0	0	0	0	0	0	1	0	1	0	0	0	0	1	0
0	0	0	0	0	1	0	1	0	0	1	0	0	0	0
0	0	0	0	0	0	1	1	0	0	0	0	1	0	0
0	0	0	0	0	0	1	1	0	0	0	0	1	0	0
0	0	0	0	0	0	0	1	1	0	0	0	0	0	1
0	0	0	0	0	1	0	0	1	0	0	1	0	0	0
0	0	0	0	0	1	0	0	1	0	0	1	0	0	0
0	0	0	0	0	0	0	0	0	1	1	0	1	0	0
0	0	0	0	0	0	0	1	1	0	0	0	0	0	1
0	0	0	0	0	1	1	0	0	1	0	0	0	0	0
0	0	0	0	0	0	0	0	0	0	0	0	1	1	1
0	0	0	0	0	0	0	0	0	1	0	1	0	1	0
0	0	0	0	0	0	0	0	0	1	1	0	1	0	0
0	0	0	0	0	0	0	0	0	0	1	1	0	0	1
0	0	0	0	0	0	0	0	0	1	0	1	0	1	0
**6**	**6**	**6**	**6**	**6**	**6**	**6**	**6**	**6**	**6**	**6**	**6**	**6**	**6**	**6**

For peer review only

For peer review only

BMJ Open

Risk of bias and reporting completeness of randomised controlled trials in burn care: Protocol for a systematic review.

Journal:	*BMJ Open*
Manuscript ID	bmjopen-2019-033472.R1
Article Type:	Protocol
Date Submitted by the Author:	11-Nov-2019
Complete List of Authors:	Young, Amber; University of Bristol, Bristol Centre for Surgical Research, Population Health Sciences, Bristol Medical School; University Hospitals Bristol NHS Foundation Trust, Paediatric Anaesthesia<br>Reeves, Barnaby; University of Bristol, School of clinical sciences<br>Cheng, Hung-Yuan; University of Bristol , Bristol Medical School<br>Wasiak, Jason; The University of Melbourne Faculty of Medicine Dentistry and Health Sciences<br>Muir, Duncan; University of Bristol Medical School<br>Davies, Anna; University of Bristol, Centre for Academic Child Health<br>Blazeby, Jane; University of Bristol, Bristol Centre for Surgical Research, Bristol Medical School
**Primary Subject Heading**:	Research methods
Secondary Subject Heading:	Evidence based practice
Keywords:	Burns, Randomised Controlled Trials as topic, Bias, Quality, CONSORT, Cochrane

SCHOLARONE™ Manuscripts

RCT quality assessment protocol Final after reviewer comments AYoung 111119

Risk of bias and reporting completeness of randomised controlled trials in burn care: Protocol for a systematic review.

Amber E Young*[1], Barnaby Reeves[2], Hung-Yuan Cheng[1], Jason Wasiak[3], Duncan Muir[1], Anna Davies[1], Jane M. Blazeby[1,4]

[1]University of Bristol, Bristol Centre for Surgical Research, Bristol Medical School, Canynge Hall, 39 Whatley Road, Bristol UK, BS8 2PS, UK.

[2] University of Bristol, School of Clinical Sciences, Level 7, Bristol Royal Infirmary, Marlborough St, Bristol, UK, BS2 8HW, UK.

[3] Faculty of Medicine, Dentistry and Health Sciences, Melbourne School of Health Sciences, The University of Melbourne, Victoria, Australia.

[4] NIHR Biomedical Research Centre at University Hospitals Bristol NHS Foundation Trust and the University of Bristol UK.

*Corresponding author: Dr Amber E Young

Tel: 0117 3427910 email: amber.young1@nhs.net

Word count: 1,813

For peer review only

For peer review only

Abstract

Introduction: Burn care represents a healthcare and economic burden to patients internationally. Choice of the most clinically effective treatment strategies requires evidence which is best obtained through high quality randomised controlled trials (RCT). The number of published RCTs of burn care is increasing. However, trial quality and reporting standards are unclear. This study will assess the risk of bias and adequacy of reporting in recent burn care RCTs using tools endorsed by the Cochrane Collaboration.

Methods and analysis: A systematic literature review will be undertaken, assessing parallel group RCTs evaluating therapeutic interventions for patients with cutaneous burns. Literature searches will use Ovid MEDLINE, Ovid EMBASE, Web of Science and the Cochrane Library. Separate searches for each database will include medical subject heading and free text terms including ‘burn’, ‘scald’ ‘thermal injury’ and ‘RCT’. Two reviewers will independently assess each study for inclusion. Risk of bias (RoB) will be assessed with the revised tool (RoB 2) and reporting completeness with the CONsolidated Standards of Reporting Trials (CONSORT 2010) guidelines. We will report a narrative synthesis of all

For peer review only - http://bmjopen.bmj.com/site/about/guidelines.xhtml

studies, including domain-specific, and overall risk of bias for the primary outcome of each trial. Inter-rater agreement for RoB 2 will be reported using Fleiss's Kappa. For adherence to the CONSORT guidelines, we will generate a completeness of reporting index for the five domains.

Ethics and dissemination: No ethics approval is required because published documents will be used. Findings of the study will be disseminated in a peer-reviewed journal and presented at conferences.

PROSPERO registration: CRD42018111020

Keywords

Burns; Randomised Controlled Trials as topic; Bias; quality; CONSORT; Cochrane

Strengths and Limitations:

- This systematic review will be the first to assess both the internal validity of burn care randomised controlled trials using the Risk of Bias 2 (RoB 2) tool and adherence to CONsolidated Standards of Reporting Trials (CONSORT 2010) reporting guidelines.
- The inter-rater reliability of RoB 2 will be assessed using six reviewers from different research backgrounds to simulate real world conditions.
- The systematic review will be limited to five-years and to articles published in English.
- Assessments of external validity will not be performed.

1. Introduction

For peer review only - http://bmjopen.bmj.com/site/about/guidelines.xhtml

For peer review only

In 2009, Chalmers and colleagues estimated clinical research waste to be 85% of global investment[1]. Wastage was attributed to methodological flaws in randomised controlled trials (RCTs). Poor quality RCTs may result in misleading conclusions increasing research waste [2-4]. Numbers of RCTS in burn care research are increasing, as new technologies are regularly introduced [5-9]. However, trial quality and adherence to reporting standards are uncertain[10-13]. Previous studies are limited by their lack of comprehensiveness and methods of quality assessment[14-16]. Quality assessment of RCTs involves evaluating internal and external validity. External validity assesses whether study results can be generalised to other populations[17]. Internal validity, or risk of bias (ROB), is the extent to which the study design is free from bias; a systematic error that leads to a deviation of results from the truth[18]. Transparent reporting is necessary to allow a clear assessment of the trial design and conduct as recommended by the Cochrane Collaboration [19 20].

Using an objective method to assess RoB and reporting completeness has become increasingly common and considered good practice [18 21]. For assessment of RoB, many tools are available, including overall scores and checklists[22 23]. The Cochrane collaboration endorse a different type of tool based on individual domains[18]. RoB domains are assessed separately, with the overall RoB is formed relating to the highest domain score. Accumulating evidence shows that this is the best tool for assessing the ROB of RCTs[24-26]. A revised version of the tool (RoB 2) has been developed which now examines single trial outcomes, includes 'signalling questions' to aid decision-making and a method to allow an overall RoB judgement [27]. As yet, this tool has not been tested for inter-rater reliability. The Cochrane collaboration recommend 'The CONsolidated Standards of Reporting Trials (CONSORT) statement' to assess adherence to reporting standards[28].

For peer review only - http://bmjopen.bmj.com/site/about/guidelines.xhtml

The aim of this review is to assess the risk of bias and adherence to reporting standards using Cochrane-approved tools. It will also assess inter-rater reliability of the new RoB 2 tool.

2. Methods

This review will meet its aim with these objectives, it will:

- Determine the number of parallel-group, individually randomised trials assessing burn care interventions published over the last five years and retrieve included full text articles.
- Assess the internal validity of the included RCTs using the revised Cochrane-endorsed Risk of Bias (RoB 2) tool.
- Assess the inter-rater reliability for the new RoB 2 tool.
- Assess the adherence of the included RCTs to CONsolidated Standards of Reporting Trials (CONSORT 2010) reporting guidelines.

b. Literature review search strategy

The systematic review will adhere to this pre-specified protocol and the Preferred Reporting Items for Systematic Reviews and Meta-Analyses (PRISMA) statement. This protocol will be aligned to the PRISMA-P statement[29-31]. It has been registered with the PROSPERO international prospective register of systematic reviews (CRD42018111020). We will report any amendments to the protocol that occur while undertaking the study, within the final manuscript.

I. Study Eligibility

For peer review only - http://bmjopen.bmj.com/site/about/guidelines.xhtml

Types of studies: Included studies must be full text individually randomised parallel RCTs published in peer-reviewed journals limited to those allocating human subjects to an intervention or control group . We have planned to limit the search to the last five year period. We will not attempt to include all burn care RCTs, as would be undertaken in assessment of the effect of an intervention. Instead, we will investigate whether recent publications of burn care RCTs adhere to the CONSORT Statement when reporting their findings and whether the trials we include are at low risk of bias according to the revised Cochrane risk-of-bias assessment tool (ROB 2). We will exclude RCT protocols, conference proceedings, abstracts, non-English language publications, interim analysis reports, and studies not involving human subjects. Health economic evaluation reports of clinical trials will be considered if they contain enough information for assessing risk of bias (e.g. clearly described methods for the trial conduct). We will also exclude trials that compare treatments *within* subjects as there is not yet a RoB 2 tool designed for assessing such trials. Also, in burn care research, these are typically not cross-over trials as generally defined[32 33]. Instead, they commonly use two wounds or two parts of the same wound. It is uncertain what the dependence between these might be, or how treatments might "contaminate" one another.

Types of participants: We will include studies evaluating two or more interventions in patients of any age with cutaneous burns. Studies where the population consists of patients with combined burn and mechanical injuries will be excluded, as the data relating to burn patients alone is likely to be difficult to disaggregate.

Type of interventions: Interventions to treat cutaneous burns of any aetiology.

For peer review only - http://bmjopen.bmj.com/site/about/guidelines.xhtml

Types of outcome: Clinical or patient-reported outcomes. Laboratory studies will be excluded.

II. Identification of studies

A pre-defined search strategy previously designed by the authors in conjunction with experienced systematic reviewers to identify RCTs in the field of burn care. Electronic searches of Ovid MEDLINE, Ovid EMBASE, Web of Science and The Cochrane Library will be searched using medical subject heading and free text terms including ‘burn’, ‘scald’ ‘thermal injury’ and ‘RCT’. To limit the search to RCTs, we will use terms derived from published RCT search strategies on Medline and the BMJ best practice guideline[34 35]. The thesaurus vocabulary of each database will be used to adapt the search terms. The search strategy for Ovid MEDLINE is included in a previous publication and in Table 1[36].

**Ovid MEDLINE search strategy**
1. Burns/ (MESH) exp
2. Burn*.tw
3. Scald*.tw
4. Thermal* adj injur*.mp
5. or/1-4
6. Heartburn.mp
7. Burnout.mp
8. (Burn* adj out).mp
9. Burning.mp
10. Burnetii.mp
11. Burnish*.mp
12. Burnet*.mp
13. 6 OR 7 OR 8 OR 9 OR 10 OR 11 OR 12
14. 5 NOT 13
15. Limit to RCT, clinical trial, English language, human, last five years

For peer review only

Table 1: Ovid MEDLINE search strategy

III. Study selection process:

Before screening abstracts and full text papers, authors undertaking study selection, RoB 2 assessment and data extraction, will undergo training to ensure a comparable understanding of the purpose of the review and the eligibility criteria. The reference management software EndNote (Endnote X8 Clarivate Analytics) will be used to compile all titles derived from the initial searches, with duplicates removed, for the review authors to screen titles and abstracts against the eligibility criteria. Screening of titles and abstracts will be completed by one reviewer. Of these, 20% will be checked by another reviewer independently (AY, DM). Any studies appearing to meet the inclusion criteria based on the abstract will be retrieved as full-text articles. Two reviewers will then read the full-text articles in their entirety to assess for eligibility, with decisions on inclusion and exclusion recorded. Reasons for exclusion will be ordered hierarchically from most to least important (Table 2) and applied to each full text paper. The most important reason for exclusion met by a paper will be recorded as the reason for exclusion. Any disagreements will be discussed with senior researchers (JMB, BR).

Duplicate
Not published within time period
Population not consisting of burn patients alone
Not a parallel group RCT
Not in English
Non-human/animal study
No full text available
Laboratory based study

For peer review only - http://bmjopen.bmj.com/site/about/guidelines.xhtml

Volunteer study
Oesophageal burns only
Ocular burns only
Anaesthetic/sedation technique only (pain management included)
Smoke inhalation injuries without an associated cutaneous burn
Diagnostic test trial
Protocol only

Table 2: Reasons for full text exclusion

For peer review only

d. Data extraction and analysis:

All studies will be assessed by two reviewers independently and then in duplicate (AY, DM), with disagreements resolved by discussion until consensus is reached, or by a senior reviewer (BR). Data will be extracted into a standardised data extraction Microsoft Excel spreadsheet which will be specifically designed for this study. It will be adapted from the RoB 2 tool to include details for the CONSORT checklist by one of the co-authors (VC). Before starting the assessment, reviewers will undergo training with the spreadsheet. The reviewers will then independently pilot test each tool on five RCTs. If there are significant differences in the application of the tools in the pilot round, additional testing will be undertaken[37].

Data extracted will include study details and research design. Study details will include author, publication year, number of sites and number of participants recruited per trial, design (full RCT, pilot study) and intervention tested. Risk of bias judgement will be reported

For peer review only - http://bmjopen.bmj.com/site/about/guidelines.xhtml

for all trial primary outcomes at a domain and overall level. Completeness of reporting will be reported for all RCTs.

I. Assessment of Risk of Bias

Included studies will be objectively assessed for internal validity using the revised risk of bias tool (RoB 2)[38]. The tool has five domains to assess bias arising from randomisation, deviations from intended interventions, missing outcome data, outcome measurement and in selection of the reported result. The assessment is specific to a single trial outcome. Categories of the overall risk of bias for the study outcome are low (risk of bias is low for all domains), some concerns (some concerns in at least one domain) and high (high risk of bias for at least one domain or some concerns for multiple domains).

Choice of outcome: Risk of bias will be assessed for the treatment effect for the primary outcome in each included trial. If this is not reported explicitly, we will use the following decision rule to select the treatment effect to report: we will assess the treatment effect for the outcome used to calculate sample size and, if this too is not reported, assess the treatment effect for the outcome named in the title, then the first reported outcome in the results[37].

II. Assessment of the inter-rater agreement of the RoB 2 tool

As the revised risk of bias tool (RoB 2) is new, measuring agreement between reviewers will help assess whether the new guidance with signalling questions has improved the reliability of the tool compared to the previous version[39-43]. To evaluate the inter-rater agreement, six independent reviewers will assess the same 30 studies allocated in a balanced incomplete blocks design (please see Appendix A), ensuring that each study is rated three times, each assessor rates 15 studies and all pairs of assessors rate six

For peer review only

studies. Inter-rater agreement will be measured for each domain of bias, and for the overall RoB judgment, by calculating Fleiss's Kappa scores[44 45]. We will categorise agreement as poor (0.00), slight (0.01-0.20), fair (0.21-0.40), moderate (0.41-0.60), substantial (0.61-0.80), or almost perfect (0.81-1.00)[26].

III. Assessment of reporting completeness

Completeness of reporting for each included RCT will be assessed using the latest revision of the CONSORT statement checklist[46]. For assessing completeness of reporting, we will calculate a reporting index defined as the percentage of items reported in each of five domains (title/abstract, introduction, methods, results and discussion), as has been done previously[47].

Patient and Public Involvement: There was no patient or public involvement in designing the study or writing up the study protocol.

Ethical issues

This study involves analysis of data from published literature and does not involve individual-level identifiable data. Given this, there were no privacy concerns that required ethical approval. Results from this study will be presented at conferences and through publication in a peer-reviewed journal.

Dissemination: we will publish the findings of this study in a peer-reviewed journal accessible to systematic review and burn care researchers.

For peer review only - http://bmjopen.bmj.com/site/about/guidelines.xhtml

Funding: This work was supported by a National Institute for Health Research Doctoral Research Fellowship DRF-2016-09-031. The views expressed are those of the author(s) and not necessarily those of the NHS, the NIHR or the Department of Health and Social Care. JMB is part-funded by the Bristol Biomedical Research Centre at the University Hospitals Bristol NHS Foundation Trust and the University of Bristol; Grant no. BRC-1215-2001. JMB is an NIHR Senior Investigator.

Disclaimer: The views expressed are those of the author(s) and not necessarily those of the NHS, the NIHR or the Department of Health and Social Care.

Author statement: AEY wrote the paper and conceived the project with the support of JMB and BR. H-YC devised the interactive study-specific data extraction spreadsheet. DM will contribute to data extraction and assisted in writing this paper. JMB, BR, JW, AD and H-YC edited and critically revised the article. All authors have read and approved the manuscript. AEY is the guarantor of the review.

Competing interests: None declared

I, the Submitting Author has the right to grant and does grant on behalf of all authors of the Work (as defined in the below author licence), an exclusive licence and/or a non-exclusive licence for contributions from authors who are: i) UK Crown employees; ii) where BMJ has agreed a CC-BY licence shall apply, and/or iii) in accordance with the terms applicable for US Federal Government officers or employees acting as part of their official duties; on a worldwide, perpetual, irrevocable, royalty-free basis to BMJ Publishing Group Ltd ("BMJ") its licensees and where the relevant Journal is co-owned by BMJ to the co-owners of the Journal, to publish the Work in BMJ Open and any other BMJ products and to exploit all rights, as set out in our licence.

For peer review only

The Submitting Author accepts and understands that any supply made under these terms is made by BMJ to the Submitting Author unless you are acting as an employee on behalf of your employer or a postgraduate student of an affiliated institution which is paying any applicable article publishing charge ("APC") for Open Access articles. Where the Submitting Author wishes to make the Work available on an Open Access basis (and intends to pay the relevant APC), the terms of reuse of such Open Access shall be governed by a Creative Commons licence – details of these licences and which Creative Commons licence will apply to this Work are set out in our licence referred to above.

For peer review only

References:

1. Chalmers I, Glasziou P. Avoidable waste in the production and reporting of research evidence. *The Lancet* 2009;374(9683):86-89.
2. Yordanov Y, Dechartres A, Porcher R, et al. Avoidable waste of research related to inadequate methods in clinical trials. *bmj* 2015;350:h809.
3. Ioannidis JP, Greenland S, Hlatky MA, et al. Increasing value and reducing waste in research design, conduct, and analysis. *The Lancet* 2014;383(9912):166-75.
4. Jüni P, Altman DG, Egger M. Assessing the quality of controlled clinical trials. *Bmj* 2001;323(7303):42-46.
5. Jeschke MG, Finnerty CC, Shahrokhi S, et al. Wound coverage technologies in burn care: novel techniques. *Journal of Burn Care & Research* 2013;34(6):612-20.
6. Selig HF, Lumenta DB, Giretzlehner M, et al. The properties of an "ideal" burn wound dressing– what do we need in daily clinical practice? Results of a worldwide online survey among burn care specialists. *Burns* 2012;38(7):960-66.
7. Jeschke MG, Herndon DN. Burns in children: standard and new treatments. *The Lancet* 2014;383(9923):1168-78. doi: 10.1016/s0140-6736(13)61093-4
8. Shahrokhi S, Arno A, Jeschke MG. The use of dermal substitutes in burn surgery: acute phase. *Wound Repair and Regeneration* 2014;22(1):14-22.
9. Young AE, Davies A, Bland S, et al. Systematic review of clinical outcome reporting in randomised controlled trials of burn care. *BMJ open* 2019;9(2):e025135.
10. Norman G, Christie J, Liu Z, et al. Antiseptics for burns. *Cochrane Database of Systematic Reviews* 2017(7)
11. Breederveld RS, Tuinebreijer WE. Recombinant human growth hormone for treating burns and donor sites. *Cochrane Database Syst Rev* 2012;12
12. Barajas-Nava LA, Lopez-Alcalde J, Roqué i Figuls M, et al. Antibiotic prophylaxis for preventing burn wound infection. *Cochrane Database of Systematic Reviews* 2013;6
13. Wasiak J, Cleland H, Campbell F, et al. Dressings for superficial and partial thickness burns. *The Cochrane Library* 2013
14. Danilla S, Wasiak J, Searle S, et al. Methodological quality of randomised controlled trials in burns care. A systematic review. *Burns* 2009;35(7):956-61.
15. Al-Benna S, Alzoubaidi D, Al-Ajam Y. Evidence-based burn care—an assessment of the methodological quality of research published in burn care journals from 1982 to 2008. *Burns* 2010;36(8):1190-95.
16. Sinha S, Yoon G, Shin W, et al. Burn clinical trials: A systematic review of registration and publications. *Burns* 2018;44(2):263-71.

For peer review only - http://bmjopen.bmj.com/site/about/guidelines.xhtml

17. Rothwell PM. External validity of randomised controlled trials:"to whom do the results of this trial apply?". *The Lancet* 2005;365(9453):82-93.
18. Higgins JP, Altman DG, Gøtzsche PC, et al. The Cochrane Collaboration's tool for assessing risk of bias in randomised trials. *Bmj* 2011;343:d5928.
19. Higgins JP, Green S. Cochrane handbook for systematic reviews of interventions: John Wiley & Sons 2011.
20. Cioffi I, Farella M. Quality of randomised controlled trials in dentistry. *International dental journal* 2011;61(1):37-42.
21. Djulbegovic B, Guyatt GH. Progress in evidence-based medicine: a quarter century on. *The Lancet* 2017;390(10092):415-23.
22. Moher D, Jadad AR, Nichol G, et al. Assessing the quality of randomized controlled trials: an annotated bibliography of scales and checklists. *Controlled clinical trials* 1995;16(1):62-73.
23. Olivo SA, Macedo LG, Gadotti IC, et al. Scales to assess the quality of randomized controlled trials: a systematic review. *Physical therapy* 2008;88(2):156-75.
24. Zeng X, Zhang Y, Kwong JS, et al. The methodological quality assessment tools for preclinical and clinical studies, systematic review and meta-analysis, and clinical practice guideline: a systematic review. *Journal of evidence-based medicine* 2015;8(1):2-10.
25. Verhagen AP, de Vet HC, de Bie RA, et al. The art of quality assessment of RCTs included in systematic reviews. *Journal of clinical epidemiology* 2001;54(7):651-54.
26. Page MJ, McKenzie JE, Higgins JP. Tools for assessing risk of reporting biases in studies and syntheses of studies: a systematic review. *BMJ open* 2018;8(3):e019703.
27. al SJe. RoB 2: a revised tool for assessing risk of bias in randomised trials. *BMJ* 2019;(in press)
28. Moher D, Hopewell S, Schulz KF, et al. CONSORT 2010 statement: updated guidelines for reporting parallel group randomised trials. *BMJ (Clinical research ed)* 2010;340:c332.
29. Moher D, Shamseer L, Clarke M, et al. Preferred reporting items for systematic review and meta-analysis protocols (PRISMA-P) 2015 statement. *Systematic reviews* 2015;4(1):1.
30. Shamseer L, Moher D, Clarke M, et al. Preferred reporting items for systematic review and meta-analysis protocols (PRISMA-P) 2015: elaboration and explanation. *Bmj* 2015;349:g7647.
31. Moher D, Stewart L, Shekelle P. Implementing PRISMA-P: recommendations for prospective authors: BioMed Central, 2016.
32. Mills EJ, Chan A-W, Wu P, et al. Design, analysis, and presentation of crossover trials. *Trials* 2009;10(1):27.
33. Sibbald B, Roberts C. Understanding controlled trials Crossover trials. *Bmj* 1998;316(7146):1719-20.
34. Glanville JM, Lefebvre C, Miles JN, et al. How to identify randomized controlled trials in MEDLINE: ten years on. *Journal of the Medical Library Association* 2006;94(2):130.
35. Study design search filters. *BMJ Best Practice*
36. Young AB, S.; Rumsey, N.; Blazeby, J. Agreement on what to measure in randomised controlled trials in burn care: study protocol for the development of a core outcome set. *Bmj Open* 2017;7(6) doi: 10.1136/bmjopen-2017-017267
37. Gates A, Gates M, Duarte G, et al. Evaluation of the reliability, usability, and applicability of AMSTAR, AMSTAR 2, and ROBIS: protocol for a descriptive analytic study. *Systematic reviews* 2018;7(1):85.
38. Higgins J, Sterne J, Savović J, et al. A revised tool for assessing risk of bias in randomized trials In: Chandler J, McKenzie J, Boutron I, Welch V (editors). Cochrane Methods. *Cochrane Database of Systematic Reviews* 2016;10
39. Armijo-Olivo S, Ospina M, da Costa BR, et al. Poor reliability between Cochrane reviewers and blinded external reviewers when applying the Cochrane risk of bias tool in physical therapy trials. *PloS one* 2014;9(5):e96920.
40. Graham N, Haines T, Goldsmith CH, et al. Reliability of 3 assessment tools used to evaluate randomized controlled trials for treatment of neck pain. *Spine* 2012;37(6):515-22.

For peer review only - http://bmjopen.bmj.com/site/about/guidelines.xhtml

41. Hartling L, Hamm MP, Milne A, et al. Testing the risk of bias tool showed low reliability between individual reviewers and across consensus assessments of reviewer pairs. *Journal of clinical epidemiology* 2013;66(9):973-81.
42. Hartling L, Ospina M, Liang Y, et al. Risk of bias versus quality assessment of randomised controlled trials: cross sectional study. *Bmj* 2009;339:b4012.
43. Bilandzic A, Fitzpatrick T, Rosella L, et al. Risk of bias in systematic reviews of non-randomized studies of adverse cardiovascular effects of thiazolidinediones and cyclooxygenase-2 inhibitors: application of a new cochrane risk of bias tool. *PLoS medicine* 2016;13(4):e1001987.
44. Hallgren KA. Computing inter-rater reliability for observational data: an overview and tutorial. *Tutorials in quantitative methods for psychology* 2012;8(1):23.
45. Nelson KP, Edwards D. A measure of association for ordered categorical data in population-based studies. *Statistical methods in medical research* 2018;27(3):812-31.
46. Schulz KF, Altman DG, Moher D. CONSORT 2010 statement: updated guidelines for reporting parallel group randomised trials. *BMC medicine* 2010;8(1):18.
47. Horsley T, Galipeau J, Petkovic J, et al. Reporting quality and risk of bias in randomised trials in health professions education. *Medical education* 2017;51(1):61-71.

For peer review only

Assessors and assigned RCTs

RCT	A	B	C	D	E	F	n
1	yes	yes	yes				3
2	yes	yes		yes			3
3	yes	yes			yes		3
4	yes	yes				yes	3
5	yes	yes	yes				3
6	yes	yes		yes			3
7	yes		yes		yes		3
8	yes			yes		yes	3
9	yes		yes		yes		3
10	yes			yes		yes	3
11	yes				yes	yes	3
12	yes				yes	yes	3
13	yes		yes	yes			3
14	yes		yes			yes	3
15	yes			yes	yes		3
16		yes		yes		yes	3
17		yes	yes		yes		3
18		yes		yes	yes		3
19		yes		yes	yes		3
20		yes			yes	yes	3
21		yes	yes			yes	3
22		yes	yes			yes	3
23			yes	yes	yes		3
24		yes			yes	yes	3
25		yes	yes	yes			3
26				yes	yes	yes	3
27			yes	yes		yes	3
28			yes	yes	yes		3
29			yes		yes	yes	3
30			yes	yes		yes	3
	**15**	**15**	**15**	**15**	**15**	**15**	

**number of RCTs:**	**30**	**#1-30**
‘n’ assessors/RCT:	3	
‘n’ assessments:	90	
‘n’ assessors:	6	A-F
‘n’ RCTs/assessor:	15	
	AB	6
	AC	6
	AD	6
	AE	6
	AF	6
	BC	6
	BD	6
	BE	6
	BF	6
	CD	6
	CE	6
	CF	6
	DE	6
	DF	6
	EF	6
		90

RCTs assessed by all pairs of assessors

RCT	AB	AC	AD	AE	AF	BC	BD	BE	BF	CD	CE	CF	DE	DF	EF
1	1	1	0	0	0	1	0	0	0	0	0	0	0	0	0
2	1	0	1	0	0	0	1	0	0	0	0	0	0	0	0
3	1	0	0	1	0	0	0	1	0	0	0	0	0	0	0
4	1	0	0	0	1	0	0	0	1	0	0	0	0	0	0
5	1	1	0	0	0	1	0	0	0	0	0	0	0	0	0
6	1	0	1	0	0	0	1	0	0	0	0	0	0	0	0
7	0	1	0	1	0	0	0	0	0	0	1	0	0	0	0
8	0	0	1	0	1	0	0	0	0	0	0	0	0	1	0
9	0	1	0	1	0	0	0	0	0	0	1	0	0	0	0
10	0	0	1	0	1	0	0	0	0	0	0	0	0	1	0
11	0	0	0	1	1	0	0	0	0	0	0	0	0	0	1
12	0	0	0	1	1	0	0	0	0	0	0	0	0	0	1
13	0	1	1	0	0	0	0	0	0	1	0	0	0	0	0
14	0	1	0	0	1	0	0	0	0	0	0	1	0	0	0
15	0	0	1	1	0	0	0	0	0	0	0	0	1	0	0
16	0	0	0	0	0	0	1	0	1	0	0	0	0	1	0
17	0	0	0	0	0	1	0	1	0	0	1	0	0	0	0
18	0	0	0	0	0	0	1	1	0	0	0	0	1	0	0
19	0	0	0	0	0	0	1	1	0	0	0	0	1	0	0
20	0	0	0	0	0	0	0	1	1	0	0	0	0	0	1
21	0	0	0	0	0	1	0	0	1	0	0	1	0	0	0
22	0	0	0	0	0	1	0	0	1	0	0	1	0	0	0
23	0	0	0	0	0	0	0	0	0	1	1	0	1	0	0
24	0	0	0	0	0	0	0	1	1	0	0	0	0	0	1
25	0	0	0	0	0	1	1	0	0	1	0	0	0	0	0
26	0	0	0	0	0	0	0	0	0	0	0	0	1	1	1
27	0	0	0	0	0	0	0	0	0	1	0	1	0	1	0
28	0	0	0	0	0	0	0	0	0	1	1	0	1	0	0
29	0	0	0	0	0	0	0	0	0	0	1	1	0	0	1
30	0	0	0	0	0	0	0	0	0	1	0	1	0	1	0
	**6**	**6**	**6**	**6**	**6**	**6**	**6**	**6**	**6**	**6**	**6**	**6**	**6**	**6**	**6**

For peer review only

For peer review only - http://bmjopen.bmj.com/site/about/guidelines.xhtml

For peer review only

PRISMA-P (Preferred Reporting Items for Systematic review and Meta-Analysis Protocols) 2015 checklist: recommended items to address in a systematic review protocol*

Section and topic	Item No	Checklist item	
**ADMINISTRATIVE INFORMATION**			
Title:			
Identification	1a	Identify the report as a protocol of a systematic review	p1
Update	1b	If the protocol is for an update of a previous systematic review, identify as such	N/A
Registration	2	If registered, provide the name of the registry (such as PROSPERO) and registration number	p1
Authors:			
Contact	3a	Provide name, institutional affiliation, e-mail address of all protocol authors; provide physical mailing address of corresponding author	p1
Contributions	3b	Describe contributions of protocol authors and identify the guarantor of the review	p7
Amendments	4	If the protocol represents an amendment of a previously completed or published protocol, identify as such and list changes; otherwise, state plan for documenting important protocol amendments	p4
Support:			
Sources	5a	Indicate sources of financial or other support for the review	p7
Sponsor	5b	Provide name for the review funder and/or sponsor	p7
Role of sponsor or funder	5c	Describe roles of funder(s), sponsor(s), and/or institution(s), if any, in developing the protocol	
**INTRODUCTION**			
Rationale	6	Describe the rationale for the review in the context of what is already known	p2-3
Objectives	7	Provide an explicit statement of the question(s) the review will address with reference to participants, interventions, comparators, and outcomes (PICO)	p4
**METHODS**			
Eligibility criteria	8	Specify the study characteristics (such as PICO, study design, setting, time frame) and report characteristics (such as years considered, language, publication status) to be used as criteria for eligibility for the review	p4
Information sources	9	Describe all intended information sources (such as electronic databases, contact with study authors, trial registers or other grey literature sources) with planned dates of coverage	p5
Search strategy	10	Present draft of search strategy to be used for at least one electronic database, including planned limits, such that it could be repeated	Table 1
Study records:			
Data management	11a	Describe the mechanism(s) that will be used to manage records and data throughout the review	p5

For peer review only

For peer review only - http://bmjopen.bmj.com/site/about/guidelines.xhtml

Selection process	11b	State the process that will be used for selecting studies (such as two independent reviewers) through each phase of the review (that is, screening, eligibility and inclusion in meta-analysis)	p5
Data collection process	11c	Describe planned method of extracting data from reports (such as piloting forms, done independently, in duplicate), any processes for obtaining and confirming data from investigators	p5
Data items	12	List and define all variables for which data will be sought (such as PICO items, funding sources), any pre-planned data assumptions and simplifications	p5-6
Outcomes and prioritization	13	List and define all outcomes for which data will be sought, including prioritization of main and additional outcomes, with rationale	p5-6
Risk of bias in individual studies	14	Describe anticipated methods for assessing risk of bias of individual studies, including whether this will be done at the outcome or study level, or both; state how this information will be used in data synthesis	p6
Data synthesis	15a	Describe criteria under which study data will be quantitatively synthesised	p6
	15b	If data are appropriate for quantitative synthesis, describe planned summary measures, methods of handling data and methods of combining data from studies, including any planned exploration of consistency (such as I^2, Kendall's τ)	
	15c	Describe any proposed additional analyses (such as sensitivity or subgroup analyses, meta-regression)	
	15d	If quantitative synthesis is not appropriate, describe the type of summary planned	
Meta-bias(es)	16	Specify any planned assessment of meta-bias(es) (such as publication bias across studies, selective reporting within studies)	
Confidence in cumulative evidence	17	Describe how the strength of the body of evidence will be assessed (such as GRADE)	

*** It is strongly recommended that this checklist be read in conjunction with the PRISMA-P Explanation and Elaboration (cite when available) for important clarification on the items. Amendments to a review protocol should be tracked and dated. The copyright for PRISMA-P (including checklist) is held by the PRISMA-P Group and is distributed under a Creative Commons Attribution Licence 4.0.**

From: Shamseer L, Moher D, Clarke M, Ghersi D, Liberati A, Petticrew M, Shekelle P, Stewart L, PRISMA-P Group. Preferred reporting items for systematic review and meta-analysis protocols (PRISMA-P) 2015: elaboration and explanation. BMJ. 2015 Jan 2;349(jan02 1):g7647.

For peer review only
